# Loss of RPS27a expression regulates the cell cycle, apoptosis, and proliferation via the RPL11-MDM2-p53 pathway in lung adenocarcinoma cells

**DOI:** 10.1186/s13046-021-02230-z

**Published:** 2022-01-24

**Authors:** Hongyan Li, Hong Zhang, Guomin Huang, Zhitong Bing, Duling Xu, Jiadi Liu, Hongtao Luo, Xiaoli An

**Affiliations:** 1 Department of Medical Physics, Institute of Modern Physics, Chinese Academy of Sciences, Lanzhou, 730000 China; 2 Gansu Provincial Isotope Laboratory, Lanzhou, 730300 China; 3 Key Laboratory of Heavy Ion Radiation Biology and Medicine of Chinese Academy of Sciences, Lanzhou, 730000 China; 4 Key Laboratory of Basic Research on Heavy Ion Radiation Application in Medicine, Lanzhou, 730000 China; 5 School of Nuclear Science and Technology, University of Chinese Academy of Sciences, Beijing, 101408 China; 6 Advanced Energy Science and Technology Guangdong Laboratory, Huizhou, 516029 China; 7 University of Chinese Academy of Sciences, Beijing, 100039 China; 8 Department of Computational Physics, Institute of Modern Physics, Chinese Academy of Sciences, Lanzhou, 730000 China; 9 Department of Radiation Medicine, Institute of Modern Physics, Chinese Academy of Sciences, Lanzhou, 730000 China; 10 Radiation Oncology of Gansu Provincial Cancer Hospital, Lanzhou, 730030 China

**Keywords:** Ribosomal protein S27a (RPS27a), Lung adenocarcinoma, Apoptosis

## Abstract

**Background:**

Depletion of certain ribosomal proteins induces p53 activation, which is mediated mainly by ribosomal protein L5 (RPL5) and/or ribosomal protein L11 (RPL11). Therefore, RPL5 and RPL11 may link RPs and p53 activation. Thus, this study aimed to explore whether RPs interact with RPL11 and regulate p53 activation in lung adenocarcinoma (LUAD) cells.

**Methods:**

The endogenous RPL11-binding proteins in A549 cells were pulled down through immunoprecipitation and identified with a proteomics approach. Docking analysis and GST-fusion protein assays were used to analyze the interaction of ribosomal protein S27a (RPS27a) and RPL11. Co-immunoprecipitation and in vitro ubiquitination assays were used to detect the effects of knockdown of RPS27a on the interaction between RPS27a and RPL11, and on p53 accumulation. Cell cycle, apoptosis, cell invasion and migration, cell viability and colony-formation assays were performed in the presence of knockdown of RPS27a. The RPS27a mRNA expression in LUAD was analyzed on the basis of the TCGA dataset, and RPS27a expression was detected through immunohistochemistry in LUAD samples. Finally, RPS27a and p53 expression was analyzed through immunohistochemistry in A549 cell xenografts with knockdown of RPS27a.

**Results:**

RPS27a was identified as a novel RPL11 binding protein. GST pull-down assays revealed that RPS27a directly bound RPL11. Knockdown of RPS27a weakened the interaction between RPS27a and RPL11, but enhanced the binding of RPL11 and murine double minute 2 (MDM2), thereby inhibiting the ubiquitination and degradation of p53 by MDM2. Knockdown of RPS27a stabilized p53 in an RPL11-dependent manner and induced cell viability inhibition, cell cycle arrest and apoptosis in a p53-dependent manner in A549 cells. The expression of RPS27a was upregulated in LUAD and correlated with LUAD progression and poorer prognosis. Overexpression of RPS27a correlated with upregulation of p53, MDM2 and RPL11 in LUAD clinical specimens. Knockdown of RPS27a increased p53 activation, thus, suppressing the formation of A549 cell xenografts in nude mice.

**Conclusions:**

RPS27a interacts with RPL11, and RPS27a knockdown enhanced the binding of RPL11 and MDM2, thereby inhibiting MDM2-mediated p53 ubiquitination and degradation; in addition, RPS27a as important roles in LUAD progression and prognosis, and may be a therapeutic target for patients with LUAD.

**Graphical Abstract:**

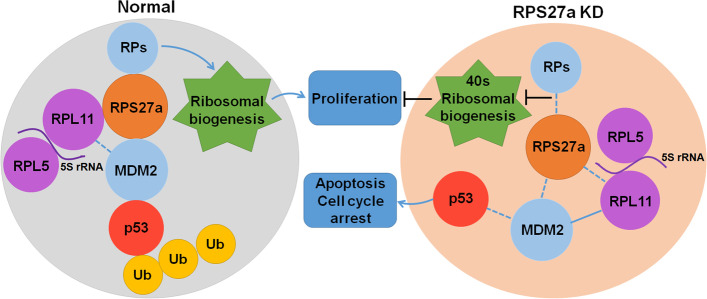

**Supplementary Information:**

The online version contains supplementary material available at 10.1186/s13046-021-02230-z.

## Background

China has the highest lung cancer incidence worldwide [1, 2]. Lung adenocarcinoma (LUAD) is the main histological subtype of lung cancer [3], and the 5-year overall survival rate of LUAD is less than 20% [4]. The mechanism of LUAD development is complex, and the effect of oncogenes on LUAD is still unknown [5]. Moreover, because expression of the tumor suppressor p53 is inhibited, p53 cannot exert transcriptional activation effects in LUAD [6]. The stability and activation of p53 are mainly regulated by murine double minute 2 (MDM2), which is part of the MDM2–p53 feedback loop necessary for regulating apoptosis [7]. MDM2-interacting proteins, including ribosomal proteins (RPs) [8] and Numb [9], also regulate p53 activation through their association with MDM2.

Under nucleolar stress, some RPs that are ribosomal subunits freely enter the nucleoplasm without being degraded by the proteasome [10]. They then directly or indirectly bind MDM2 and inhibit its E3 ubiquitin ligase activity, thereby stabilizing and activating p53 [11]. A variety of RPs bind MDM2 and form RP–MDM2–p53 pathways, such as RPS7 [12], RPL5 and RPL11 [13], and RPL26 [14]. These RPs are translocated from the nucleolus to the nucleoplasm and then regulate the p53 activity. Knockdown of RPs including RPL22 [15], RPL24 [16], RPL29 and RPL30 [17], RPL4 [18], RPS14 [17] and RPS26 [19] results in RPL5 and/or RPL11-dependent p53 activation, given that RPL5 and RPL11 are regulators of p53 activation under nucleolar stress [20]. The RPL5/RPL11–MDM2–p53 complex is the classical model of interaction between RPs and p53 [21]. RPL5 and RPL11 act with MDM2 either by themselves or in a 5S ribonucleoprotein complex with 5S rRNA [22]. Moreover, deletion of RPs including RPL29 and RPL30 [23], and RPS6 [24] induces p53 upregulation, a process mediated mainly by RPL5 and/or RPL11 binding and inhibition of MDM2. Therefore, RPL5 and RPL11 may link RPs and p53 activation through enhancing their interaction with MDM2 after deletion of RPs. In previous findings [25], we demonstrated that RPL27a interacts with MDM2 and RPL5, thereby regulating p53 activation in GC-1 cells.

In this study, we reasoned that some RPs might bind RPL5 or RPL11 and form an RP–RPL5/RPL11 complex, thereby regulating p53. Furthermore, knockdown of these RPs might enhance the interaction of RPL5 and/or RPL11 with MDM2, thus inhibiting MDM2-mediated p53 ubiquitination and leading to p53 stabilization. In this regard, we used immunoprecipitation (IP) and mass spectrometry (MS) to identify the RPs interacting with RPL11. Among the identified RPs, the ribosomal protein S27a (RPS27a) was notably identified as a novel regulator of the RPL11–MDM2–p53 pathway. RPS27a was highly expressed in patients with LUAD, thus indicating that RPS27a expression might be associated with LUAD progression. The knockdown of RPS27a induced p53-dependent cell cycle arrest, apoptosis and inhibition of cell viability in A549 cells, in a manner dependent on RPL11. The present study revealed that RPS27a directly bound RPL11, and RPS27a knockdown enhanced the binding of RPL11 and MDM2, thereby inhibiting MDM2-mediated p53 ubiquitination and degradation in A549 cells.

## Methods

### Cell culture and transient transfection

We prepared four cell lines (BeNa, Culture Collection, Beijing, China), BEAS-2B (The human bronchial epithelial cells, no.BNCC254518), H460 (The human large cell lung cancer cell line, no. BNCC233991), A549 (The human non-small-cell lung cancer cell lines, no. BNCC290808) and H1299 (The human non-small-cell lung cancer cell lines, no. BNCC334400), they were cultured in F-12 K medium (Hyclone, MA, USA) with 10% fetal bovine serum (Gibco, CA, USA) and kept under 37 °C with 5% CO_2_. Follow-up experiments were performed when the cells were in the logarithmic phase of growth and 70% confluence. When the cells grew to 70% confluence, they were transfected with siRNAs for 24 or 48 h.

### Plasmids, drugs, antibodies, and siRNAs

His-tagged RPL11 expression plasmids were constructed by inserting the RPL11 cDNA into the pET32a His vector at *Nco*I *and Xho*I sites (Supplementary File 1). The RPL11 cDNA was amplified using the following mRNA primers: 5′-GACGACGACAAGGCCATGGCTGCGCAGGATCAAGGTG-3′ and 5′-GTGGTGGTGGTGGTGCTCGAGTTTATTTGCCAGGAAGGATG-3′. A GST-RPS27a *Escherichia coli* expression vector was constructed by inserting the RPS27a cDNA into the pEGX-6P-1 vector at *Bam*HI and *Xho*I sites (Supplementary File 1). The RPS27a cDNA was amplified using the following mRNA primers: 5′-GTTCCAGGGGCCCCTGGGATCCATGCAGATTTTCGTGAAAAC-3′ and 5′-CAGTCACGATGCGGCCGCTCGAGTTACTTGTCTTCTGGTTTG-3′. The overexpression of Flag-tagged RPS27a plasmids were constructed by inserting the pEX-3-RPS27a cDNA into the pcDNA3.1-3xFlag-C vector at *XbaI* and *HindIII* sites (Supplementary File 1). The pEX-3-RPS27a cDNA was amplified using the following mRNA primers: 5′-GCTCTAGATTACTTGTCTTCTGGTTTGT-3′ and 5′-CCCAAGCTTATGCAGATTTTCGTGAAAAC-3′. GST-RPS27a, His-RPL11 and Flag-RPS27a were also generated with polymerase chain reaction (PCR) and cloned into the vector.

Lipo2000 (no. 11668019, Invitrogen, CA, USA) was used for transient transfection. β-actin (no. ab8227), RPS27a (no. ab74731), RPL11 (no. ab74731), p53 (no. ab74731), p21 (no. ab109199), Nucleolin (no. ab129200) and E-adherin (no. ab40772) (Abcam, Cambridge, UK), MDM2 (no. ab16895) (Genetex, NJ, USA), Nucleophosmin (no. sc-32256) (Santa Cruz Biotechnology, CA, USA), Ki-67 (no. GB111141) and MMP-9 (no. GB11132) (Servicebio biotechnology, Wuhai, China) were used for analysis of immunoblotting (IB), immunofluorescence and immunohistochemistry. MG132 (no. HY-13259) and Cycloheximide (CHX, no. HY-12320) were purchased from Medchemexpress (NJ, USA), Doxorubicin (Dox, no. GC16994) and Actinomycin D (ActD, no. GC16866) were purchased from Glpbio (CA, USA). The IC_50_ of doxorubicin (Dox) on A549 cells and its effect on the survival of A549 cell clones are shown in Supplementary File 2. Three different sequences of siRNA of each gene was synthesized by Genepharma (Shanghai, China); their sequences were shown in Supplementary File 3.

### Immunoprecipitation and mass spectrometry (IP/MS)

IP of RPL11 was performed as described previously [26]. Briefly, A549 cells in logarithmic growth phase were collected and lysed, and an equal amount of lysate was used for IB and IP analyses. Then, 10 μg of rabbit RPL11 antibodies and the same amount of rabbit IgG (Beyotime, Shanghai, China) were added to lysates from the experimental and control groups, and incubated overnight at 4 °C. After elution and purification, the immunoprecipitates were separated by SDS-PAGE, then silver stained. The bands of binding proteins were digested topeptides and then analyzed with an LC-MS/MS (TripleTOF, AB Sciex, Boston, MA, USA) instrument, and the results were evaluated. Credibility ≥95% and unique peptides ≥1 were the criteria used to identify proteins [27].

### Immunoblotting

The cells were lysed with RIPA buffer (Beyotime), and total protein was separated with 10% SDS-PAGE gels and then transferred to polyvinylidene difluoride (PVDF) membrances (Millipore, Bedford, MA, USA). The membranes were blocked with tris-buffered saline with tween 20 (TBST) containing 5% skim milk powder and incubated with primary antibodies at 4 °C overnight. After secondary antibody binding, an chemiluminescence reagents kit (New cell & Molecular Biotech, Suzhou, China) was used to detect protein bands. β-actin was used as a control, and the intensity of protein bands was analyzed in AlphaView SA software (Alpha Innotech, CA, USA).

### GST-fusion pull down assay

His-tagged RPL11 expression plasmids were transfected in *Escherichia coli BL21* (*E. coli*)*.* His-RPL11 was purified with an Ni^2+^-NTA column (Thermo Fisher Scientific, MA, USA) after expression in *E. coli.* GST-fusion assays were conducted as previously described [28]. Briefly, 50 μg GST-RPS27a or GST was mixed with glutathione Sepharose 4B beads (Sigma, MO, USA) and incubated with 20 μg purified His-RPL11 proteins. Then, anti-S-Tag and GST antibodies were used to analyze protein interactions by IB.

### Co-immunoprecipitation (co-IP) analyses and in vitro ubiquitination assay

For the co-immunoprecipitation (co-IP) assays, A549 cells were transfected with Flag-tagged RPS27a or vector control, then lysed. Subsequently, 70% of the lysate was incubated with anti-Flag monoclonal antibody (Cell signaling technology, USA) or control IgG, and the remaining 30% of the lysate was analyzed with IB. In vitro ubiquitination experiments followed protocols from previous studies using the Ni^2+^-NTA purification method. The A549 cells were transfected with His-Ub plasmids after transfection of RPS27a-siRNA for 24 h, then treated with 40 μM MG132 for 6 h. Subsequently, 70% of the lysate was incubated anti-His monoclonal antibody (Cell signaling technology) and used for ubiquitination experiments with co-IP assays; the bead-bound proteins and the other 30% of the lysate were analyzed with IB [29].

### Immunofluorescence and immunohistochemistry

Immunofluorescence assays were performed as described previously [26]. Briefly, after permeabilization, blocking with 5% bovine serum albumin (BSA) in tris-buffered saline (TBS), incubation with a primary antibody (1:100) overnight at 4 °C and staining with 5 μg/mL 4′, 6-diamidino-2-phenylindole (DAPI), the cells were covered with coverslips and then scanned with a confocal laser microscope (LSM, Carl Zeiss AG, Germany) or observed under a biomicroscope (BX53, Olympus, Tokyo, Japan).

The LUAD samples and xenograft tumors were analyzed by immunohistochemistry, as previously described [30]. Paraffin-embedded tissues were cut into 4-μm thick sections, deparaffinized with xylene and dehydrated in ethanol, incubated in 3% hydrogen peroxide, blocked with TBST containing 10% (v/v) BSA and incubated with primary antibody at 4 °C overnight. Then, the secondary antibody was added to the sections, and protein expression was detected with 3, 3′-diaminobenzidine. Finally, the sections were counterstained with hematoxylin, then scanned with Panoramic MIDI software (3DHISTECH, Budapest, Hungary). Image-Pro Plus software was also used to analyze the optical density of protein expression.

### Cell cycle and apoptosis analysis

After 48 h of transfection, A549 cells were collected and performed for cell cycle and apoptosis analyses. Briefly, 50 μg/mL of propidium iodide (PI) (Meilune, Dalian, China) was used to stain suspended cells at 37 °C in the dark for 30 min. Data on DNA content were collected with Cell Quest and analyzed in the ModFit software program. An Annexin V/PI kit (Meilune) was used to distinguish the apoptotic cells stained by 5 μL of Annexin V and 1 μL of PI for 15 min at room temperature. The apoptotic cells were analyzed with a Flow sight imaging flow cytometer (Amnis/Merck Millipore, Darmstadt, Germany).

### Reverse transcription and quantitative PCR analyses

TaKaRa company designed and synthesized primers (Dalian, China), and SYBR green dye on the StepOnePlus™ Real-time PCR System (Applied Biosystem, MA, USA). The DDCt method was used to analyze the expression levels of target genes in different groups. The primers used were 5′- AGAAGAAGTCTTACACCACTCCC-3′ and 5′- TGCCATAAACACCCCAGC-3′ (RPS27a); 5′-TCCACTGCACAGTTCGAGGG-3′ and 5′-AAACCTGGCCTACCCAGCAC-3′ (RPL11); 5′- CGACTGTGATGCGCTAATGG-3′ and 5′-AAATCTGTCAGGCTGGTCTGC-3′ (p21); 5′-CTCACCATCATCACACTGGAA-3′ and 5′-TCATTCAGCTCTCGG AACATC-3′ (p53); 5′-AATCATCGGACTCAGGTACATC-3′ and 5′-CTGCTACTGCTTCTTTCACAAC-3′ (MDM2); 5′-TCAAGAAGGTGGTGAAGCAGG-3′ and 5′-TCAAAGGTGGAGGAGTGGGT -3′ (GAPDH) [31].

### Dissociation of ribosomal subunits and measurement of the subunit ratio

Sucrose gradient sedimentation was used to analyze the ribosomal profiles as described previously [32, 33]. Briefly, 5–50% sucrose density gradient solution [20 mM HEPES (pH 7.5), 100 mM KCl, 10 mM MgCl_2_ and 200 g/mL heparin] was added to the lysates of A549 cells as separated samples. Samples were measured at 254 nm absorbance (Biocomp, CA, USA), and quantitative analysis of ribosome peaks was performed. The area under the curve for the lowest points of the 40S, 60S and 80S peaks was calculated by summing the digital measurements.

### Stably knockdown of RPS27a cells constructed

A cell line with stable knockdown of RPS27a was generated with lentiviral short hairpin (shRNA) and drug screening. A549 cells were transfected with shRNA lentiviral transfection plasmids (pLKD-CMV-EGFP-2A-Puro-U6-shRPS27a) constructed by insertion of the shRPS27a into the pLKD-CMV-EGFP-2A-Puro-U6 virus vector between the *EcoRI* and *AgeI* sites (Obio Biotech, Shanghai, China) (Supplementary File 4). The shRNA sequence of RPS27a was as follows: 5′-GTGCCCTTCTGATGAATGT-3′. Lentivirus lacking the shRNA insert was used as a negative control (pLKD-CMV-EGFP-2A-Puro-U6-NC). A suspension of 7.5 × 10^4^ cells/mL was generated with A549 cells, and 2 mL of the suspension per well was seeded in a six-well plate. The virus was added 20 h after seeding of the cells, and the cells in each plate were transfected with shRNA-RPS27a (6.26 × 10^8^ TU/mL) or shRNA-NC lentivirus (3.44 × 10^8^ TU/mL). The cells were imaged under a fluorescence microscope and further selected with puromycin with a final concentration of 2 μg/mL 72 h after lentiviral infection. Then, fresh medium with 2 μg/mL puromycin was replaced every 2–3 days for screening the A549 cells with stable knockdown of RPS27a. The cells were imaged under a fluorescence microscope again after 14 d of transfection and collected to verify the RPS27a expression with real-time PCR and IB.

### Cell viability assay

A cell counting kit 8 (CCK-8, Meilune) was used to detect cell viability. A549 and H1299 cells were seeded in 96-well plates for incubation 24 h (Corning Costar, SNY, USA) and transfected with siRNAs. Then, 10 μL of the CCK-8 reaction solution was added to the wells after transfection for 24 h and incubated at 37 °C for 4 h. A microplate reader (Tecan M200, Switzerland) was used to measure the absorbance at a wavelength of 450 nm. The formula cell viability = [A (compound +)–A (blank)]/[A (compound–)–A (blank)] was used to calculate the growth ratio.

### Colony-formation assay

The colony-formation assays on A549 cells were performed as described previously [34]. Briefly, the cells grew to 70% confluence, 30 nM Dox was added and incubated 24 h, and the cells were then digested with 0.25% trypsin. A total of 300 A549 cells were seeded on a 35-mm culture dish, and incubation continued for 14 days. The cells were then washed twice with phosphate buffered solution (PBS), fixed with methanol and stained with 0.2% crystal violet. Each group was assayed in triplicate, and the number of colonies was observed and counted.

### Transwell cell invasion and migration assay

Transwell chambers (Corning Costar) were used for Transwell invasion assays, as previously described [35]. A549 cells (3 × 10^5^/300 μL) were seeded on the upper chambers of Transwell plates coated with Matrigel matrix containing complete growth medium for the invasion assay, whereas plates without Matrigel in the upper chamber were used for the migration assay. A 500 μL volume of complete medium was added to the lower chamber after cultivation of 12 h. Simultaneously, the upper complete medium was replaced with serum-free medium, and 30 nM Dox was added into the upper chamber. The incubation continued for 24 h, and the cells on the surface of the lower chamber were fixed with 4% paraformaldehyde and stained with 0.1% crystal violet solution for cell counting. At least six randomly selected fields were counted, and the average number was presented.

### Docking analysis

Briefly, the protein-protein interaction module of Schrodinger software (Schrodinger 2015 suit) was used for analysis of RPL11 and PRS27a interactions. The three-dimensional crystal structures of the human 80S ribosome (PDBID: 4v6x) were extracted from the PDB database. The small-molecule 3D structures were docked from the X-ray crystal structures of RPL11 and PRS27a, and two proteins were extracted from the 80S ribosome. The ubiquitin and water molecules were removed from the two protein structures to simulate the interaction [36].

### Tumor xenografts

The management and handling of animals complied with the administrative regulations of the Laboratory Animal Affairs Administration of the Ministry of Science and Technology of China (1988.11.14). The research on experimental animals was approved by the Ethics Committee of the Institute of Modern Physics, Chinese Academy of Sciences, and the Institutional Animal Care and Use Committee. Five weeks-old nude mice (female, weight 16–17 g, SPF level) were obtained from the Laboratory Animal Center of GemPharmatech (Nanjing, China). NC and RPS27a knockdown cells in logarithmic growth phase were injected (2 × 10^6^) subcutaneously into the mice to establish a cell xenograft model. Tumor volume was calculated using the following equation: Tumor volume = Length × Width^2^/2. The average tumor volume of each group was calculated and expressed in mm^3^ [37].

### Statistical analysis

Graphpad prism 8.0 software (GraphPad Software, CA, USA) was used to analyze the data. Statistical differences were analyzed based on the Student’s *t*-test and on one-way analysis of variance test with Turkey. The results were expressed as the mean ± S.D. Correlation analysis was calculated with a Spearman’s and Pearson’s correlation coefficient in SPSS/PC program (Version 19.0; SPSS Inc., Chicago, IL, USA). A *p* value of < 0.05 was considered statistically significant. Clinical data of gene were calculated by Kaplan-Meier survival curves, and the groups were compared using the log-rank test.

## Results

### RPS27a is a potential binding protein with RPL11

**Fig. 1:**
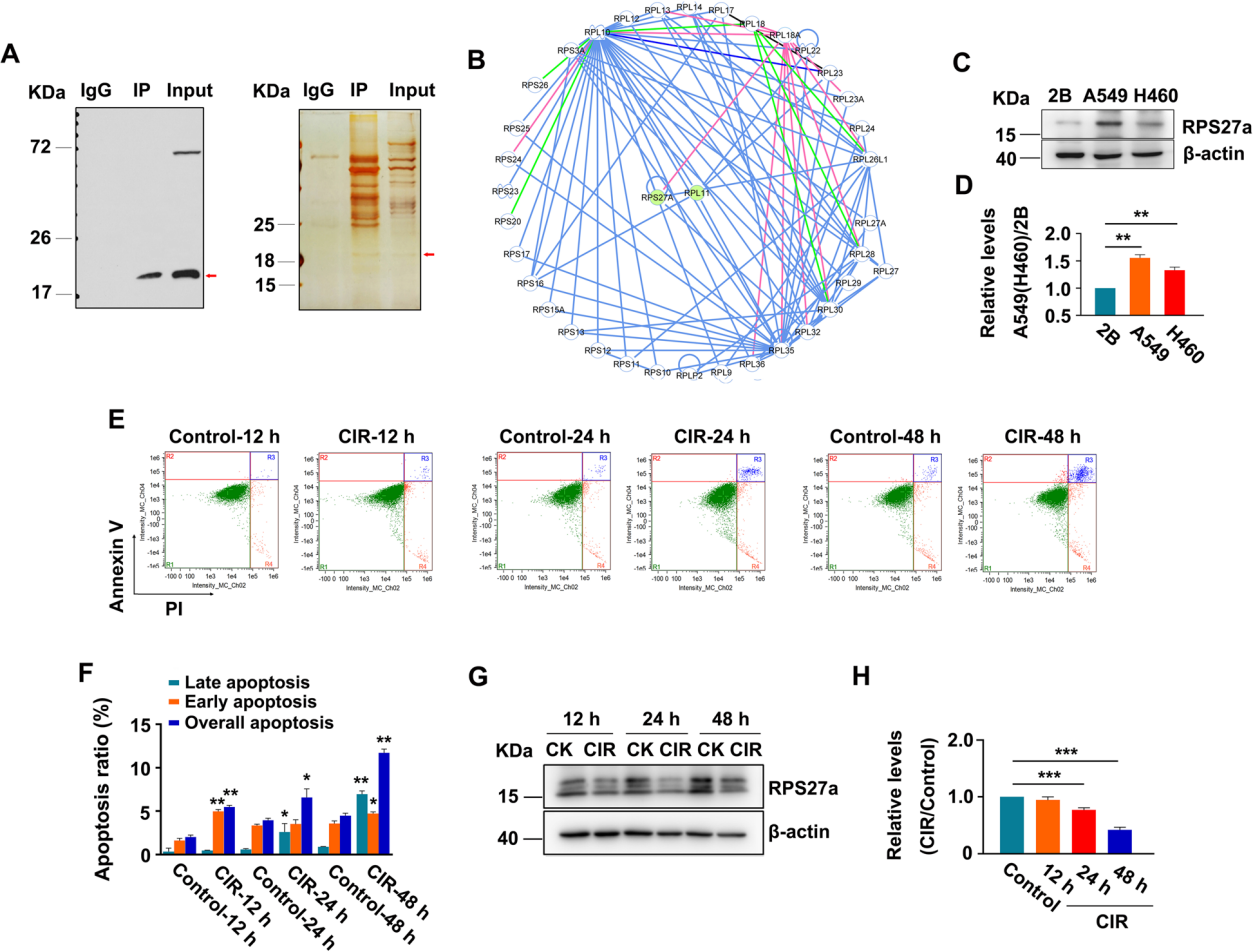
Identification of RPs interacting with RPL11 in A549 cells. **A** The endogenous RPL11-interacting proteins were pulled down by anti-RPL11 antibody. The immunoprecipitates were separated by SDS-PAGE, then silver stained. The band (approximately 18 kDa) containing proteins strongly bound to RPL11 was digested with trypsin and analyzed with LC-MS/MS. **B** Interaction network of RPs with RPL11, on the basis of the STRING database. **C** and **D** The expression of RPS27a was analyzed by IB in BEAS-2B, A549 and H460 cells. The expression of RPS27a was quantified (RPS27a/β-actin), the normalized RPS27a in BEAS-2B cells was set at 1.0, and **p < 0.01 was calculated with ANOVA (*n* = 3) (**D**). **E** and **F** Apoptosis of A549 cells was analyzed by flow cytometry at 12, 24 and 48 h after CIR. R1, main population; R2, necrotic cells; R3, late apoptotic cells; R4, early apoptotic cells. Total apoptotic cells = R3 + R4. The percentages of apoptosis after CIR. *p < 0.05, **p < 0.01 were calculated with *t*-test (*n* = 3) (**F**). **G** IB analysis of RPS27a expression in A549 cells after CIR. **H** The expression of RPS27a was quantified (RPS27a/β-actin), and the normalized RPS27a in control cells at 12, 24 and 48 h after CIR was set at 1.0. ***p < 0.001 were calculated with the *t*-test (*n* = 3). CK, control; CIR, carbon ion radiation; IB, immunoblotting; 2B, BEAS-2B

The untreated A549 cell lysate was used to identify endogenous RPL11-binding proteins through IP/MS to discover potential RPL11-binding RPs. The silver stained image of the binding proteins revealed a band at approximately 18 kDa (Fig. 1A). The band was further analyzed by MS, and a total of 133 proteins were identified in the IP protein sample. The protein-related information is shown in Supplementary File 5, among which 43 interactors were RPs (Fig. 1B). The combined degree of RPS27a was highest. Next, we detected the expression of RPS27a in BEAS-2B, A549 and H460 cells (Fig. 1C). The expression of RPS27a in A549 and H460 cells was higher than that in BEAS-2B cells and was highest in A549 cells (Fig. 1D), thus, indicating that the overexpression of RPS27a was associated with the progression of non-small cell lung cancer. Moreover, a previous study has shown that RPS27a is involved in the regulation of p53 levels. Therefore, we focused on RPS27a, according to the hypothesis that the RPS27a-RPL11 interaction might play a role in p53 activation.

### Correlation of A549 cell apoptosis with RPS27a expression

Inducing tumor cell apoptosis is a common strategy to inhibit tumor development. We demonstrated that carbon ion radiation (CIR)-induced nucleolar stress decreases RPL27a expression and promotes spermatogonia apoptosis [1]. Therefore, 4 Gy CIR, a common experimental dose [1], was used to induce apoptosis of A549 cells. Then, the increased apoptosis of A549 induced by CIR was observed (Fig. 1E, F) and the decreased expression of RPS27a were time dependent (Fig. 1G, H). The correlation analysis suggested that the RPS27a level was related to the late apoptotic ratio after CIR (Supplementary File 6). Therefore, A549 cell apoptosis may be associated with decreased RPS27a expression, and we were able to induce apoptosis of A549 cells by decreasing RPS27a expression.

### Knockdown of RPS27a activated p53, promoted cell apoptosis, induced cell cycle arrest, and inhibited cell viability

**Fig. 2:**
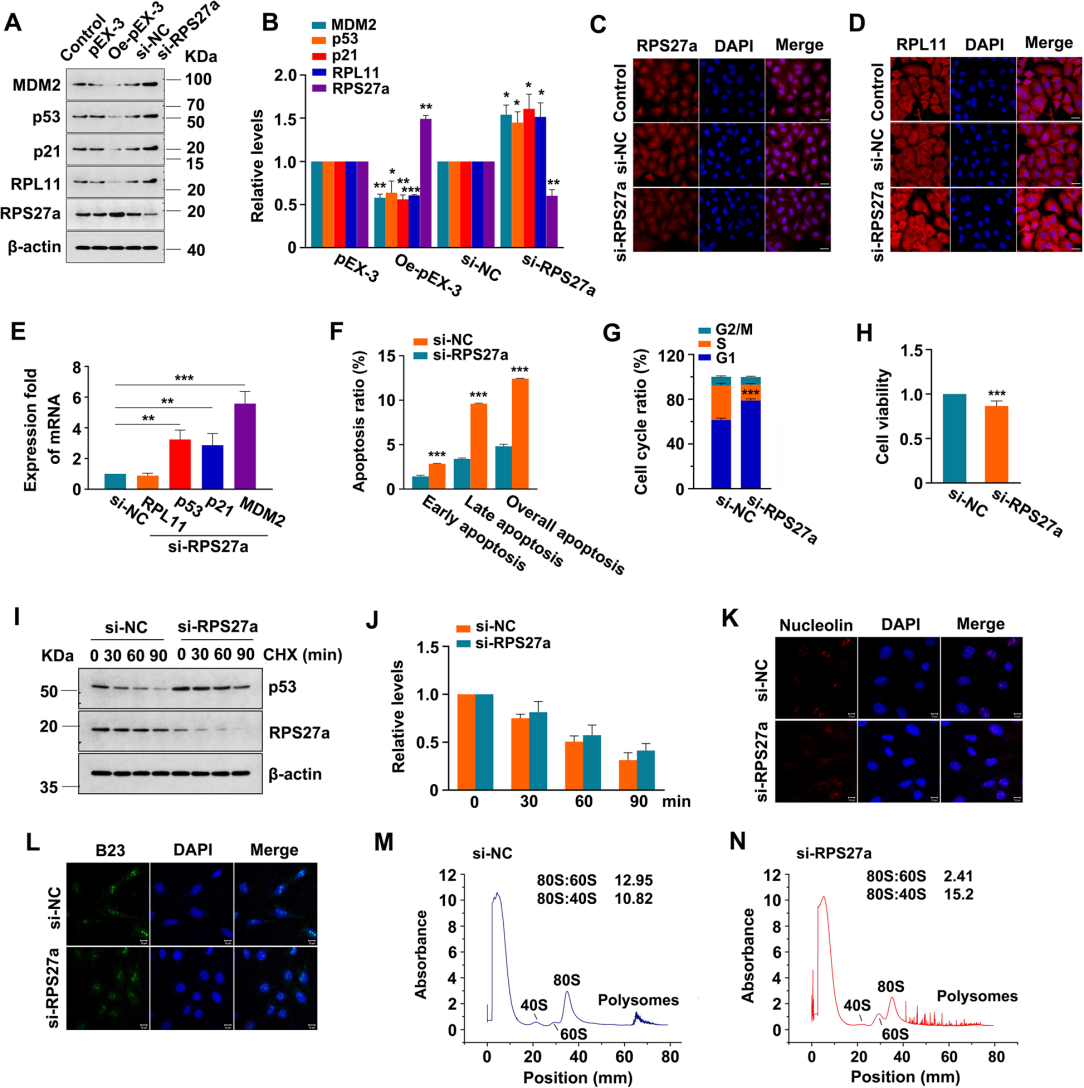
Knockdown of RPS27a stabilizes and activates p53, causes G1-phase arrest, induces apoptosis and inhibits the viability of A549 cells. **A** and **B** A549 cells transfected with RPS27a-siRNA or RPS27a overexpression plasmid (pEX-3). The protein levels were detected with IB (**A**). The expression of proteins was quantified (target protein/β-actin), and the normalized target protein in NC or vector cells was set at 1.0. *p < 0.05, **p < 0.01, **p < 0.001 were calculated with ANOVA (*n* = 3) (**B**). **C** and **D** IF of A549 cells stained with RPL11 and RPS27a antibodies; DAPI staining shows the nucleoli (magnification, 400×, bar = 50 μm). **E** The expression of mRNA levels was analyzed by real-time PCR.**p < 0.01, ***p < 0.001 were calculated with ANOVA (*n* = 3). **F**-**L** A549 cells were transfected with RPS27a-siRNA for 48 h. The percentage of apoptosis was analyzed with flow cytometry, ***p < 0.001 was calculated with the *t*-test (*n* = 3) (**F**). The cell cycle was analyzed with flow cytometry, ***p < 0.001 was calculated with the *t*-test (*n* = 3) (**G**). The cell viability was measured with the CCK-8 assay, ***p < 0.001 was calculated with the *t*-test (*n* = 5) (**H**). **I** and **J** After transfection for 48 h with RPS27a-siRNA, A549 cells were treated with 50 μg/mL CHX for 30, 60 or 90 min. The expression of p53 and RPS27a protein was analyzed with IB (**I**). The normalized p53 at time 0 min was set at 1.0 in NC and RPS27a-siRNA-treated cells (*n* = 3) (**J**). **K**-**N** A549 cells were transfected with RPS27a-siRNA for 48 h. IF analysis of the location of nuclelolin (red) and B23 (green) in A549 cells. The staining was observed with a confocal laser microscope (magnification, 400×, bar = 10 μm, blue indicates nucleoli) (**K** and **L**). Sucrose gradient sedimentation was used to analyze the ribosomal profiles; the value of ribosomal sedimentation was measured by monitoring of A254; peaks showing 40S, 60S, 80S and polysome contents are indicated (**M** and **N**). NC, negative control; Oe, overexpression; CHX, cycloheximide; IB, immunoblotting; IF, immunofluorescence; DAPI, 4', 6-diamidino-2-phenylindole; CHX, Cycloheximide

RNA interference and overexpression plasmids were used to knock down and induce overexpression of RPS27a, respectively, to explore the relationship of RPS27a and RPL11 with p53 activation. The efficiency of knockdown of RPS27a, RPL11 and p53 by three different siRNAs is shown in Fig. S1. The protein levels of p53, MDM2, p21 and RPL11 were higher in RPS27a siRNA–treated cells than NC cells (Fig. 2A, B). Similar to the immunoblotting results, the immunofluorescence results showed that the fluorescence signal of RPL11 (Fig. 2D) was enhanced in the nucleoli and cytoplasm after knockdown of RPS27a. The mRNA expression levels of p53, p53 target genes MDM2 and p21 were higher in RPS27a siRNA–treated cells than NC cells (Fig. 2E). In addition, the knockdown of RPS27a promoted cell apoptosis (Fig. 2F), increased G1-phase arrest (Fig. 2G) and inhibited cell viability (Fig. 2H). Moreover, the increased p53 expression was relatively stable, with an increased half-life (Fig. 2I, J). The aforementioned findings suggested that knockdown of RPS27a stabilized and activated p53, thus increasing G1-phase arrest and apoptosis, and inhibiting cell viability in A549 cells. Representative images of the flow cytometry results are shown in Figs. S2 and S3.

### Knockdown of RPS27a decreased small ribosomal subunits ratio in A549 cells

Immunofluorescence was used to analyze the localization of nucleolar integrity marker proteins, nucleolin (NCL) (Fig. 2K) and nucleophosmin (B23) [39] (Fig. 2L) to further determine whether RPS27a knockdown might destroy the nucleolar integrity of A549 cells. In RPS27a-siRNA cells, compared with NC cells, the B23 and NCL remained dispersed in nuclear clusters, thus indicating that knockdown of RPS27a did not disrupt the nucleoli in A549 cells. Furthermore, polysome profiles were compared to study the effects of RPS27a knockdown on the ratios between small and large ribosomal subunits in A549 cells (Fig. 2M, N). The 80S:60S ratio was diminished, and the 80S:40S ratio was elevated, in the RPS27a-siRNA cells. Thus, the knockdown of RPS27a impaired the ribosomal profiles in A549 cells, inhibited 40S ribosome biogenesis and altered the ribosomal subunit ratio.

### Knockdown of RPS27a induces p53-dependent cell cycle arrest and RPL11-dependent p53 activation in A549 cells

**Fig. 3:**
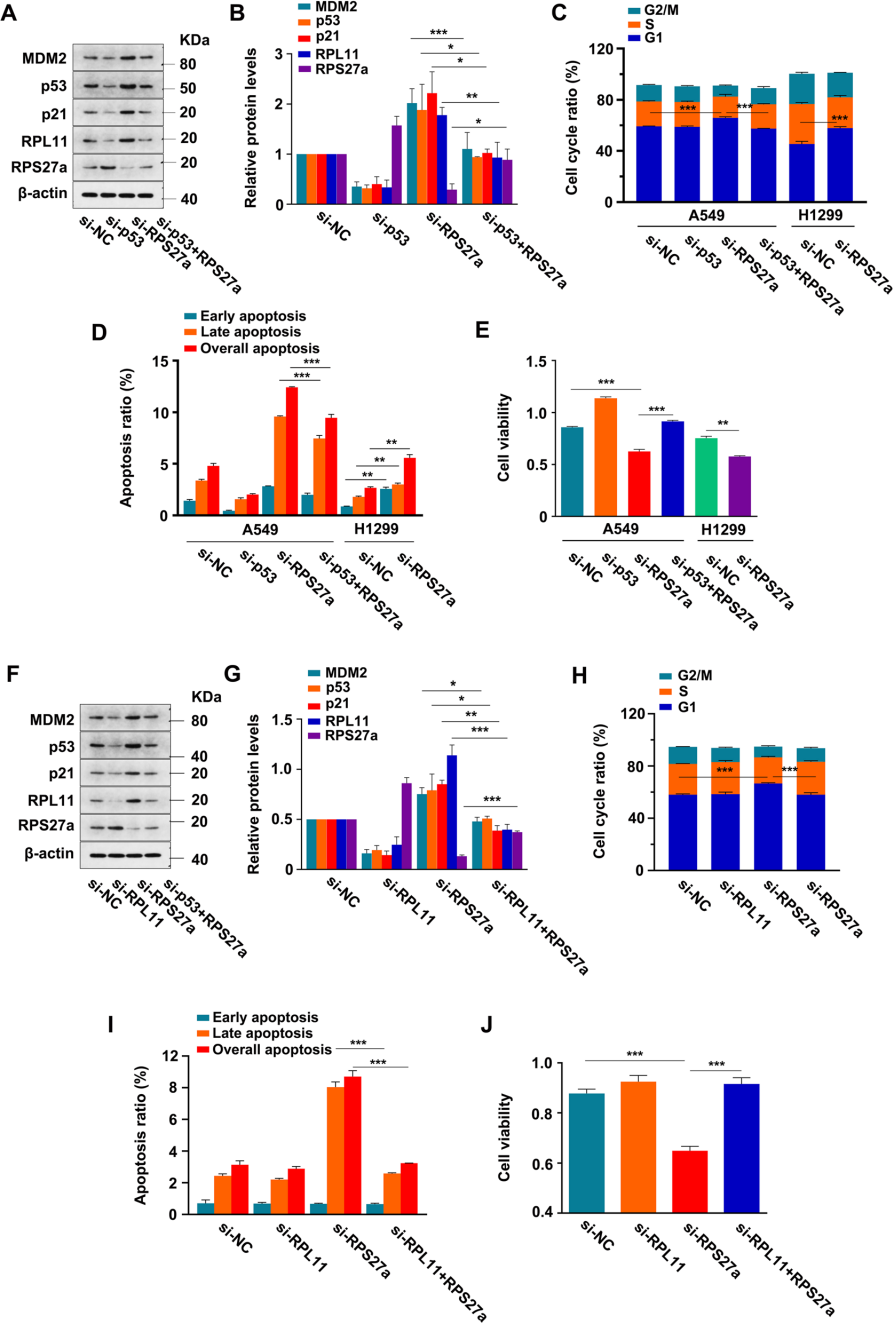
Knockdown of RPS27a induces p53-dependent cell cycle arrest and RPL11-dependent p53 activation in A549 cells. **A**-**E** A549 cells were co-transfected with p53 and RPS27a-siRNA, and H1299 cells were transfected with RPS27a-siRNA. The expression of proteins was detected with IB (**A**). The expression of proteins was quantified (target protein/β-actin), and the normalized target protein in NC cells was set at 1.0. *p < 0.05, **p < 0.01 and ***p < 0.001 were calculated with the *t*-test between co-transfected cells and cells transfected with RPS27a-siRNA alone (*n* = 3) (**B**). The cell cycle percentages were analyzed with flow cytometry. **p < 0.01, **p < 0.001 were calculated with ANOVA in A549 cells (*n* = 3). **p < 0.01 was calculated with the *t*-test in H1299 cells (*n* = 3) (**C**). The percentage of cell apoptosis was analyzed with flow cytometry. ***p < 0.001 was calculated with the *t*-test between co-transfected cells and cells transfected with RPS27a-siRNA alone (*n* = 3). **p < 0.01 was calculated with the *t*-test in H1299 cells (*n* = 3) (**D**). The cell viability was measured with CCK-8 assays. ***p < 0.001 was calculated with ANOVA in A549 cells (*n* = 5). **p < 0.01 was calculated with the *t*-test in H1299 cells (*n* = 5) (**E**). **F**-**J** A549 cells were co-transfected with RPL11 and RPS27a-siRNA. The expression of proteins was quantified (target protein/β-actin), and the normalized target protein in NC cells was set at 1.0 (*n* = 3). *p < 0.05, **p < 0.01 and ***p < 0.001 were calculated with *t*-test between co-transfected cells and cells transfected with RPS27a-siRNA alone (*n* = 3) (**F**). The cell cycle percentages were analyzed with flow cytometry. ***p < 0.001 were calculated with ANOVA in A549 cells (*n* = 3) (**G**). The percentage of cell apoptosis was analyzed with flow cytometry. ***p < 0.001 was calculated with the *t*-test between co-transfection and transfected with RPS27a-siRNA alone (*n* = 3) (**H**). The cell viability was measured with CCK-8 assays. ***p < 0.001 was calculated with ANOVA in A549 cells (*n* = 5) (**I**). NC, negative control; IB, immunoblotting

The RPS27a-siRNA and p53-siRNA co-transfection experiment showed that the knockdown of p53 eliminated the increase in MDM2 and p21 protein levels (Fig. 3A, B), G1-phase arrest (Fig. 3C) and apoptosis (Fig. 3D) induced by the knockdown of RPS27a in A549 cells. In addition, the knockdown of p53 eliminated the inhibition of cell viability in A549 cells (Fig. 3E). Interestingly, the knockdown of RPS27a also increased G1-phase arrest (Fig. 3C), moderately increased apoptosis (Fig. 3D) and suppressed cell viability in p53-deficient H1299 cells (Fig. 3E). These results suggested that RPS27a plays a critical role in the cell viability, apoptosis and cell cycle progression in a p53-dependent manner in wild type A549 cells. Representative images of the flow cytometry data are shown in Figs. S4 and S5.

Deletion of certain RPs induces ribosomal stress and p53 activation, which is mainly mediated by RPL11 [17]. RPS27a and RPL11 co-transformation experiments were performed to demonstrate that the activation of p53 under RPS27a knockdown was also regulated by this mechanism. The knockdown of RPL11 eliminated the increase in MDM2 and p21 protein levels (Fig. 3F, G), G1-phase arrest (Fig. 3H) and apoptosis (Fig. 3I) induced by the knockdown of RPS27a. In addition, knockdown of RPL11 eliminated the inhibition of cell viability (Fig. 3J). Representative images of the flow cytometry data are shown in Figs. S6 and S7. Therefore, the knockdown of RPS27a requires RPL11 to induce p53 upregulation and a decrease in cell proliferation.

### RPS27a interacts with RPL11

**Fig. 4:**
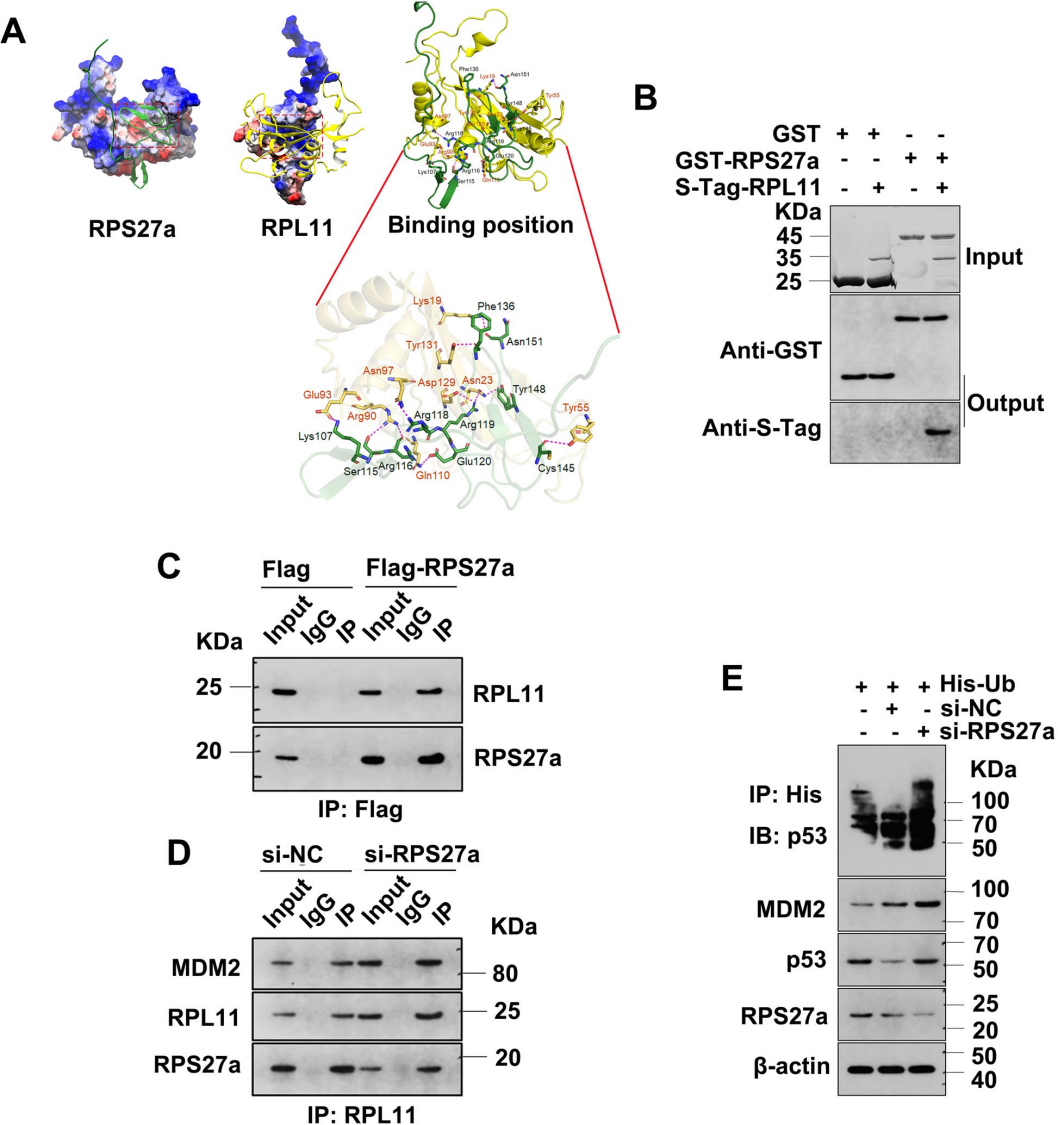
RPS27a directly binds RPL11, and the knockdown of RPS27a regulates p53 activation. **A** Docked positions of RPS27a and RPL11, shown as a cartoon model in light green and yellow. The ligands are represented as sticks in magenta, cyan and yellow-green. **B** Binding analysis of RPS27a and RPL11 in vitro with GST pull-down assays. Fusion protein beads were used for pull-down and detected with IB with anti-GST and anti-S-Tag antibodies; Coomassie staining of GST and GST-RPS27a proteins is shown in the upper panel; IB analysis is shown in the middle and lower panels. **C** A549 cells were transfected with Flag-RPS27a plasmids and harvested for co-IP assays with anti-Flag antibody. **D** A549 cells were transfected with RPS27a-siRNA for 48 h and treated with 40 μM MG132 for 4 h. The cell lysates were subjected to IP with anti-RPL11 antibody, followed by IB with antibodies to MDM2, RPS27a and RPL11. **E** A549 cells were transfected with His-Ub plasmids and RPS27a-siRNA, then treated with 40 μM MG132 for 4 h. The cell lysateswere subjected to IP with anti-His antibody, followed by IB with anti-p53 antibody to detect ubiquitinated p53. NC, negative control; IP, immunoprecipitation; co-IP, co-immunoprecipitation; IB, immunoblotting

The small-molecule 3D structures were docked from the X-ray crystal structure of RPS27a and RPL11 (Fig. 4A). RPS27a and RPL11 interactions were simulated with the protein-protein interaction module in Schrodinger software (Schrodinger 2015 suite). The 3D crystal structures of the human 80S ribosome (PDBID: 4v6x) were extracted from the PDB database (http://www.rcsb.org/). The structure and function of proteins were closely associated with the hydrogen bonding between amino acids. The well-known nucleotide-binding residues are shown in (Fig. 4A). The results of in silico docking suggested an interaction between RPS27a and RPL11.

To further confirm that RPS27a directly interacted with RPL11 in vitro, we performed GST-fusion protein–protein association assays with His-RPL11 and GST-RPS27a fusion proteins purified from bacteria. Purified His-RPL11 was bound by purified GST-RPS27a protein but not GST alone (Fig. 4B; comparison of lane 4 with lane 2 in lower panel). These results demonstrated that RPS27a directly bound RPL11 in cells.

### Knockdown of RPS27a stabilizes p53

To determine whether changes in the expression of RPS27a might affect the interaction of RPS27a and RPL11, we constructed Flag-RPS27a overexpression plasmids and transfected them into A549 cells, then performed co-IP and IB. The overexpression of RPS27a enhanced the binding of RPS27a and RPL11 (Fig. 4C; comparison of lane 6 with lane 3). This result further confirmed that RPS27a binds RPL11, and overexpression of RPS27a promotes their binding.

Next, we examined the binding between MDM2 and RPL11 after knocking down RPS27a. RPL11 readily pulled down MDM2 in co-IP assays, and our analysis indicated that the interaction between RPS27a and RPL11 was weakened (Fig. 4D; lane 6 compared with lane 3 in lower panel), but RPL11 and MDM2 was enhanced after RPS27a knockdown (Fig. 4D; lane 6 compared with lane 3 in upper panel). Therefore, the decrease in RPS27a was likely to weaken the interaction between RPS27a and RPL11, but to enhance the binding of RPL11 and MDM2, thereby inhibiting MDM2 E3 ubiquitin ligase activity and stabilizing p53.

To test the effect of RPS27a knockdown on p53 ubiquitination, we generated A549 cells transfected with His-Ub plasmids and siRNA of RPS27a to analyze p53 ubiquitination and demonstrate that RPS27a knockdown inhibits MDM2-mediated p53 ubiquitination. RPS27a knockdown increased the expression of MDM2 and p53 (Fig. 4E; comparison of lane 3 with lane 2 in lower panels), MDM2 ubiquitinated p53, whereas RPS27a knockdown inhibited this ubiquitination that led to p53 accumulation (Fig. 4E; comparison of lane 3 with lane 2 in upper panel). Therefore, RPS27a knockdown stabilizes p53 by inhibiting MDM2-mediated p53 ubiquitination and degradation.

### Knockdown of RPS27a has minimal effects on p53 protein level and stability under stress

**Fig. 5:**
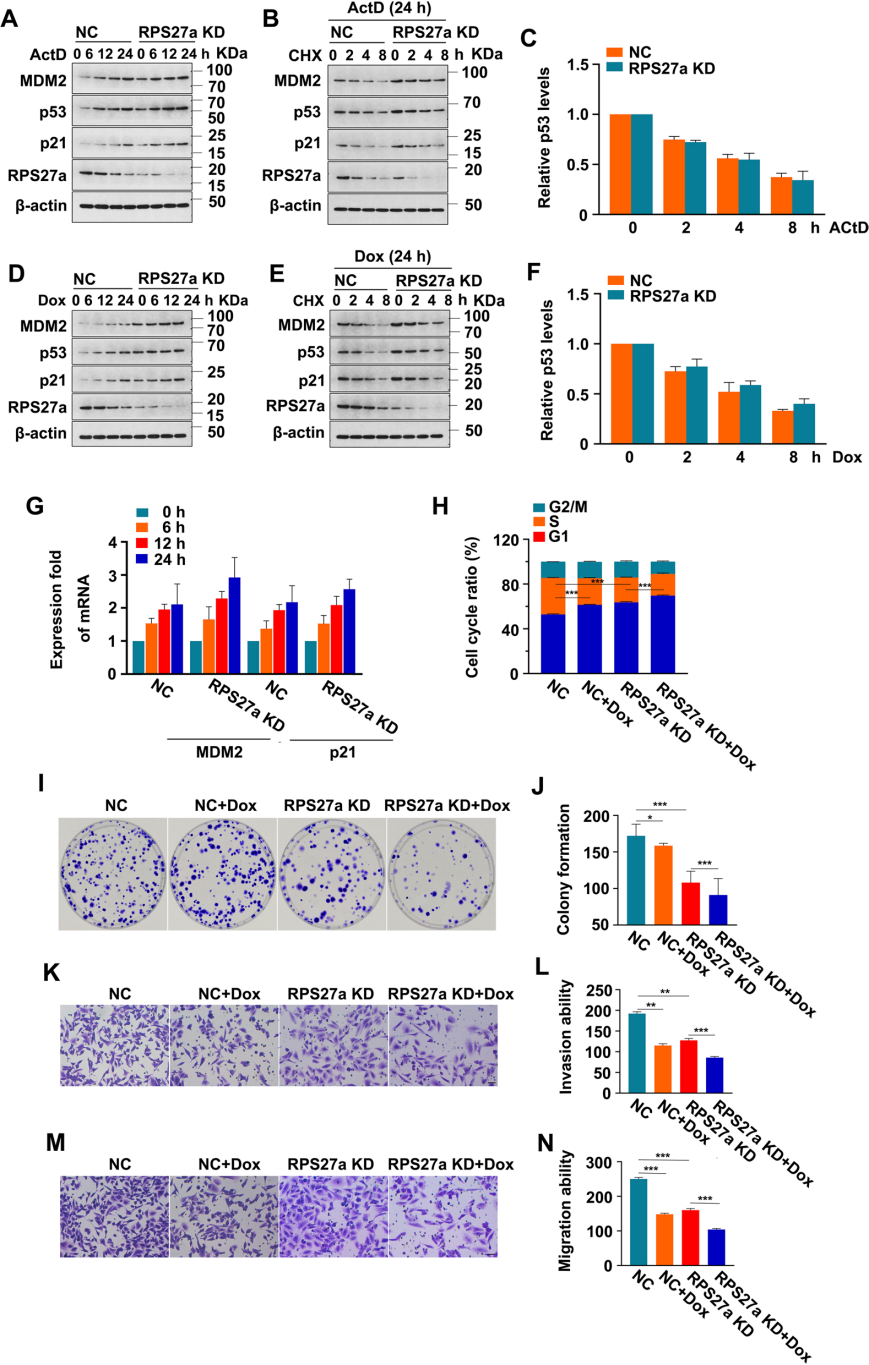
Knockdown of RPS27a regulates p53 activity without affecting its stability in response to stress. **A** NC and RPS27a knockdown of A549 cells were treated with 5 nM ActD for 6, 12 or 24 h; the protein expression was then detected with IB. **B**, **C** The NC and RPS27a knockdown A549 cells were treated with 5 nM ActD for a total of 24 h together with 50 μg/mL CHX for the indicated time periods; protein expression was then detected with IB. The expression of p53 in ActD and CHX-treated cells was quantified (p53/β-actin) and normalized to p53 at time 0 h; values were set at 1.0 for NC and RPS27a knockdown cells (*n* = 3) (**C**). **D** NC and RPS27a knockdown A549 cells were treated with 30 nM Dox for 6, 12 or 24 h, and the protein expression was detected with IB. **E** NC and RPS27a knockdown A549 cells were treated with 30 nM Dox for a total of 24 h together with 50 μg/mL CHX for the indicated time periods; the protein expression was then detected with IB. **F** The expression of p53 in Dox and CHX-treated cells was quantified (p53/β-actin) and normalized at time 0 h, with values set at 1.0 for NC and RPS27a knockdown cells (*n* = 3). **G** NC and RPS27a knockdown A549 cells were treated with 30 nM Dox for 24 h, and the expression of mRNA levels was analyzed with real-time PCR for the indicated time points (*n* = 3). **H**-**J** NC and RPS27a knockdown A549 cells were treated with 30 nM Dox for 24 h. The cell cycle percentage was analyzed with flow cytometry. ***p < 0.001 was calculated with ANOVA (*n* = 3) (**H**). Colony formation, observed and calculated (**I**). *p < 0.05, ***p < 0.001 was calculated with ANOVA (*n* = 3) (**J**). **K**-**N** NC and RPS27a knockdown A549 cells were seeded into the Transwell chamber and then treated with 30 nM Dox for 24 h. The invasion of cells in the lower chamber was observed, and the number of cells was counted (**K**). **p < 0.01, ***p < 0.001 were calculated with ANOVA (*n* = 3) (**L**). The migration of cells in the lower chamber was observed, and the number of cells was counted (**M**). ***p < 0.001 was calculated with ANOVA (*n* = 3) (**N**). NC, negative control; ActD, dactinomycin; Dox, doxorubicin; CHX, Cycloheximide; IB, immunoblotting

Depletion of RPL23 and RPS7 had no affects on p53 response [40], however, depletion of RPS25 can attenuate the p53 response under ribosomal stress [41]. To demonstrate that RPS27a is essential for the activation of p53 in response to ribosomal stress, we constructed A549 cells with stable knockdown of RPS27a. The screening results of the A549 cells with stable knockdown of RPS27a under fluorescence microscopy are shown in Fig. S8. The mRNA and protein levels of RPS27a in the A549 cells with stable knockdown of the RPS27a are shown in Fig. S9. Exposure to low dose of ActD triggers ribosomal stress and activation of p53 [42]; thus, A549 cells with stable knockdown of RPS27a were treated with 5 nM ActD [42] and then collected at different time points for IB analysis. Knockdown of RPS27a did not impair the increased levels of p53, MDM2 and p21 induced by ActD (Fig. 5A), and had minimal effects on p53 protein level and stability (Fig. 5B), because after 24 h of ActD treatment, p53 showed similar half-life changes between negative control and RPS27a knockdown A549 cells (Fig. 5C). Therefore, the knockdown of RPS27a had no effect on p53 stabilization under ribosomal stress.

We subsequently evaluated the effect of RPS27a knockdown on DNA damage–induced p53 activation. Exposure to Dox induces DNA damage and p53 activation [43]; thus, we treated A549 cells with stable knockdown of RPS27a with Dox and then collected them at different time points for IB analysis and real-time PCR. The concentration of Dox was selected, as shown in Supplementary File 2. Knockdown of RPS27a did not impair Dox-induced p53 activation (Fig. 5D) and had minimal effects on p53 protein level and stability (Fig. 5E), because after 24 h of Dox treatment, p53 showed similar half-life changes between the negative control and RPS27a knockdown A549 cells (Fig. 5F). The PCR results showed that RPS27a knockdown did not eliminate the upregulation of MDM2 and p21 at mRNA levels at different time points of Dox treatment (Fig. 5G), thus indicating that RPS27a knockdown had little effect on p53 transactivation after Dox treatment. Therefore, these observations indicated that RPS27a did not participate in DNA damage–induced p53 stabilization. In addition, knockdown of RPS27a aggravated Dox-induced G1 phase arrest (Fig. 5H), suppression of the colony-forming (Fig. 5I, J), invasion (Fig. 5K, L) and migration ability (Fig. 5M, N) in A549 cells.

### RPS27a is a oncogene in LUAD

**Fig. 6:**
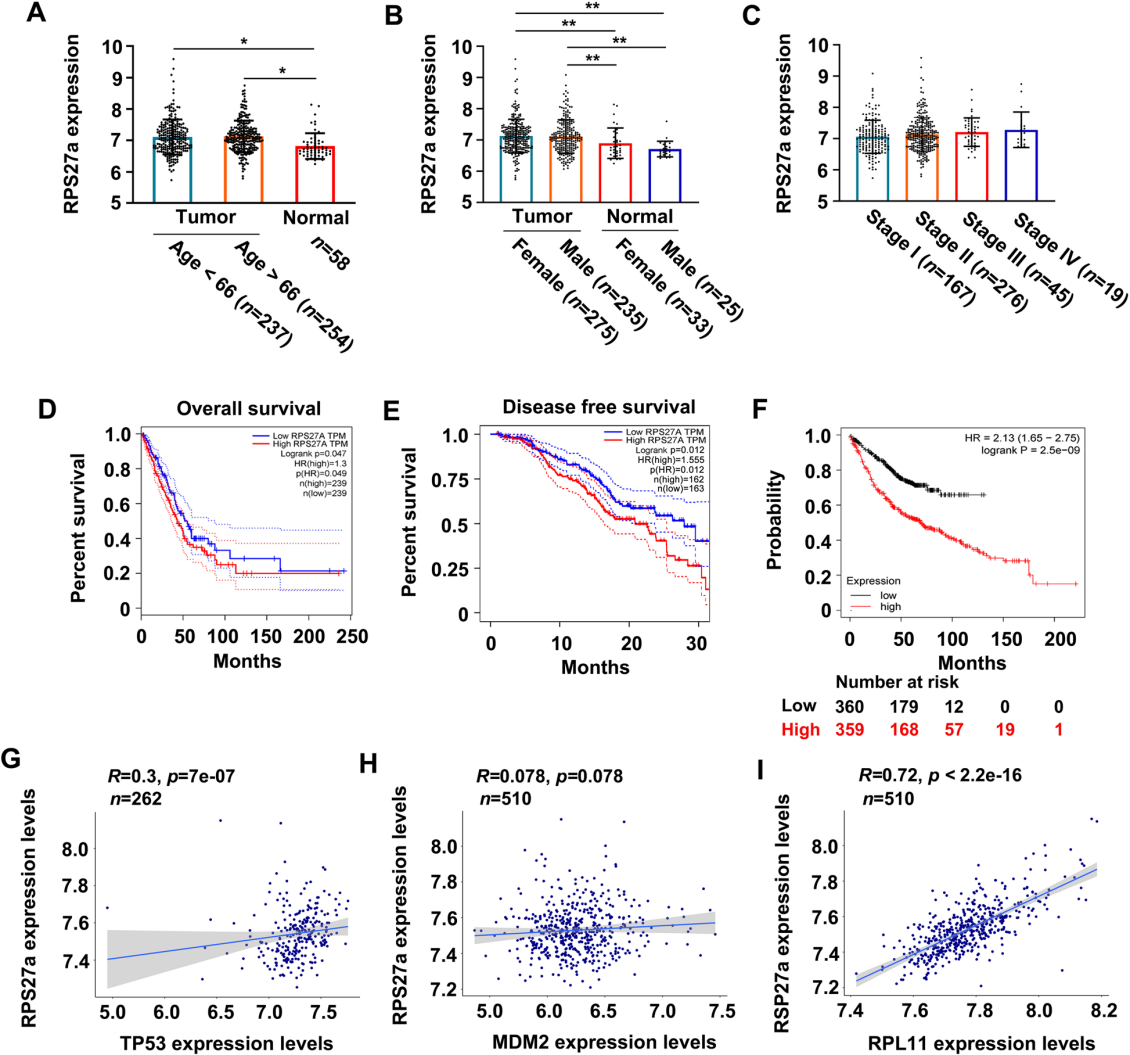
High levels of RPS27a correlate with LUAD progression and poorer prognosis. **A**, **B** Comparison of RPS27a mRNA expression between normal lung tissues and tumor tissues with LUAD from TCGA datasets. *p < 0.05, **p < 0.01 were calculated with ANOVA. **C** The expression of RPS27a mRNA in LUAD types at different stages (http://gepia.cancer-pku.cn/). **D**, **E** Kaplan-Meier curves estimating overall survival (**D**) and disease-free survival (**E**) in patients with low and high expression levels of RPS27a mRNA in LUAD from the TCGA datasets. Log-rank test, *p* < 0.001. **F** The survival in patients with low and high expression levels of RPS27a mRNA in LUAD from GEO datasets. **G**-**I** The correlation analysis of RPS27a mRNA expression between other genes in patients with LUAD from TCGA datasets using Pearson’s correlation coefficient. The expression of mRNA was converted to log value and the scatter plot was drawn by the Ggplot-R software LUAD, lung adenocarcinoma

**Fig. 7:**
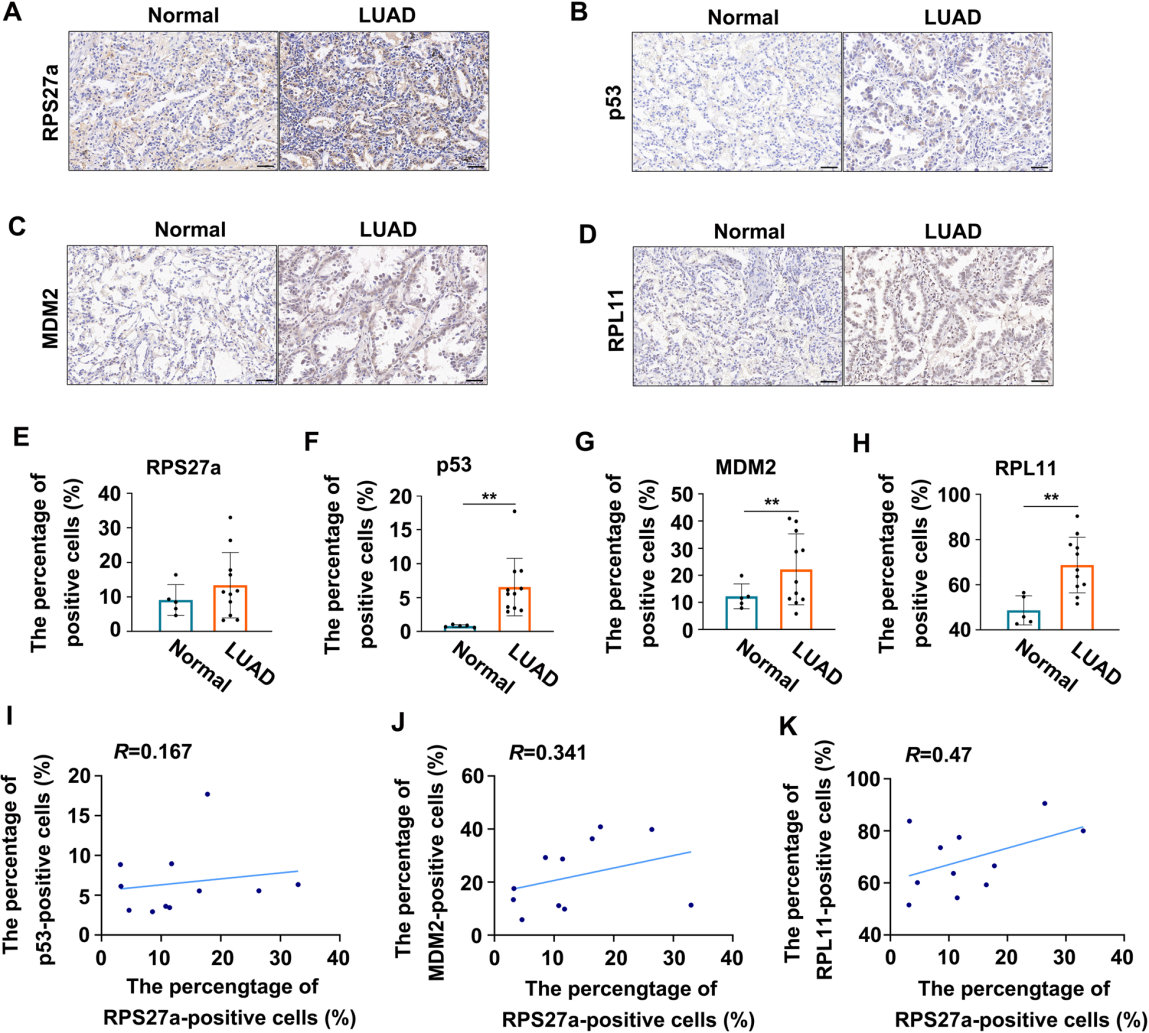
The expression and correlation analysis of RPS27a, p53, MDM2 and RPL11 protein in tumor tissues with LUAD. **A**-**D** Representative immunohistochemical images of RPS27a, p53, MDM2 and RPL11 in normal lung tissues and tumor tissues with LUAD (magnification, 200×, bar = 50 μm). **E**-**H** The percentage of RPS27a, p53, MDM2 and RPL11-positive cells in normal lung tissues and tumor tissues with LUAD. The percentage of positive cells = positive cells counting/total cells counting under 200 magnified visual field was performed with digital image analysis for quantification of proteins, six fields were randomly selected and calculated the average of positive cells in one sample. **p < 0.01 with Student’s *t*-test analysis. **I**-**K** Correlation analysis between RPS27a-positive cells and other proteins in tumor tissues with LUAD (*n* = 11) were assessed using Pearson’s correlation coefficient. LUAD, lung adenocarcinoma

The RPS27a mRNA expression in LUAD was analyzed on the basis of the TCGA dataset to determine the role of RPS27a expression in the progression of LUAD. The expression of RPS27a mRNA showed a significant difference between 491 LUAD tissues (age < 66, *n* = 237; age > 66, *n* = 254) and 58 normal tissues (Fig. 6A); a significant difference between female LUAD tissues (*n* = 275) and normal tissues (female, *n* = 33; male, *n* = 25); and a significant difference between male LUAD tissues (*n* = 235) and normal tissues (female, *n* = 33; male, *n* = 25) (Fig. 6B). However, no difference was observed in different stages and grades of LUAD (Fig. 6C). The correlation of RPS27a levels with the prognosis of patients with LUAD was evaluated on the basis of the TCGA dataset with overall and disease-free survival information. The patients were then divided into high and low RPS27a expression groups, and Kaplan-Meier survival curves were analyzed [44]. The Kaplan–Meier survival analysis showed that patients with high RPS27a expression had poorer overall survival (Fig. 6D, within 250 months) and disease-free survival (Fig. 6E, within 30 months). The survival analysis showed that high RPS27a expression was associated with poorer survival (Fig. 6F, within 200 months). These findings indicated that RPS27a expression levels significantly negatively correlated with the prognosis of patients with LUAD. In addition, the RPS27a, wild type p53, MDM2 and RPL11 mRNA expression in LUAD were analyzed on the basis of the TCGA dataset to determine the correlation between RPS27a and these three genes. The correlation analysis showed that up-regulated RPS27a mRNA is positively correlated with wild type p53, MDM2 and RPL11 mRNA expression in patients with LUAD, respectively (Fig. 6G-I). A total of 11 LUAD and 5 normal tissue specimens were collected from the Gansu Provincial Cancer Hospital to determine the correlations of RPS27a, wild type p53, MDM2 and RPL11 expression in clinical LUAD. Representative images of immunohistological staining of RPS27a are shown in Fig. 7A. To further explore the correlation of RPS27a with p53, MDM2 and RPL11 levels in patients with LUAD, we stained LUAD and normal tissue specimens for p53, MDM2 and RPL11 by IHC (Fig. 7B-D). The percentage of positive cells of RPS27a, p53, MDM2 and RPL11 protein was significantly increased in LUAD tissues compared with normal tissues, respectively (Fig. 7E-H). The correlation analysis was limited to the number of clinical samples, and showed that increased positive cells of RPS27a is probable a positive correlation with positive cells of p53, MDM2 and RPL11 protein in patients with LUAD, respectively (Fig. 7I-K). The findings provided the first demonstration that the overexpression of RPS27a in patients with LUAD might contribute to LUAD development and decreased survival, and upregulated RPS27a may positively correlated with wild type p53, MDM2 and RPL11 protein in tumor tissues with LUAD.

**Fig. 8:**
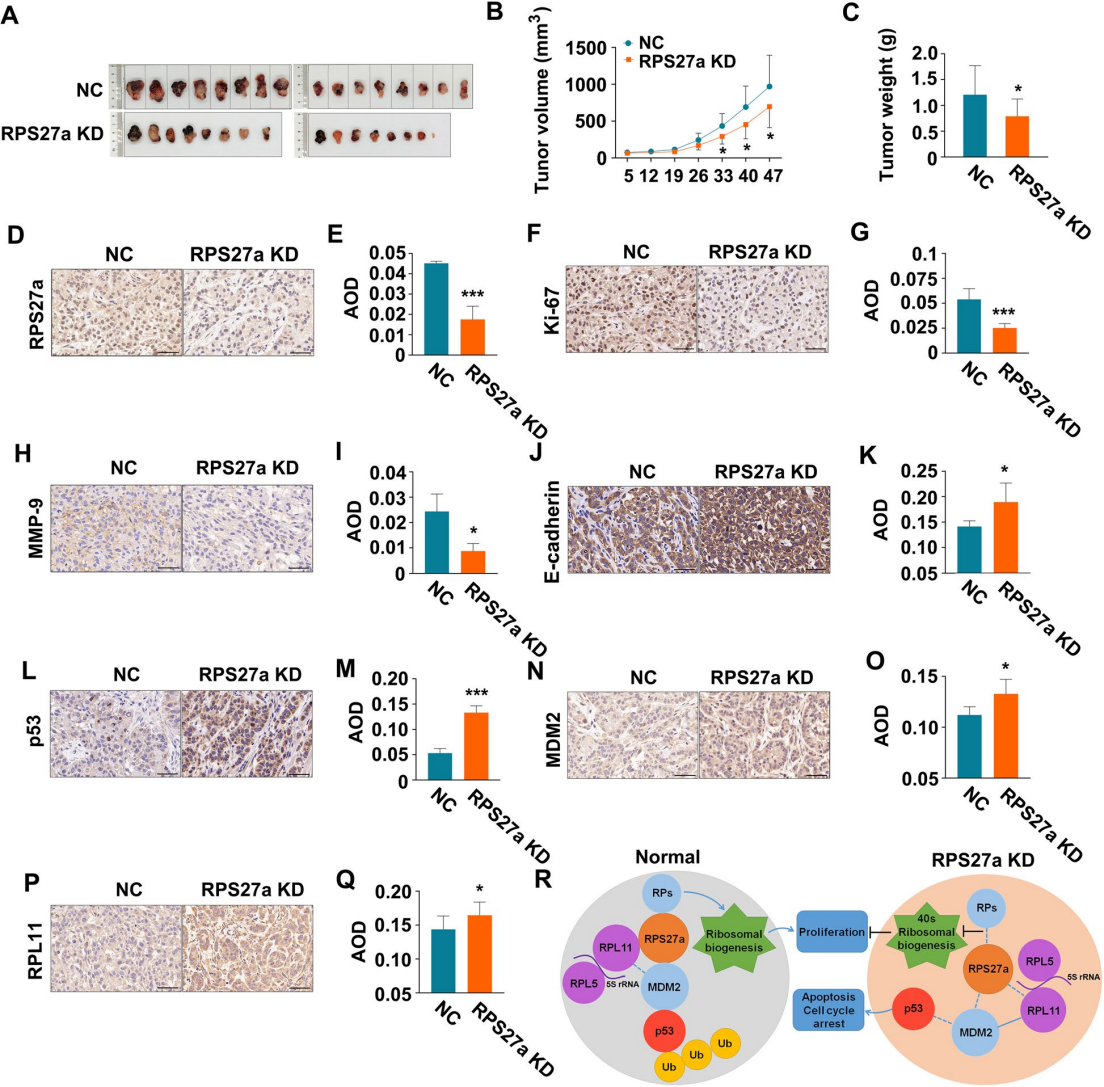
Knockdown of RPS27a activates p53 and inhibits the formation of A549 cell xenografts. **A** Images of tumors with sh-RPS27a or control cells. The mice were sacrificed 47 days after A549 cell implantation. **B** Growth curves of subcutaneous xenograft tumors from sh-RPS27a (*n* = 15) or control (*n* = 16) cells. Tumor size was calculated every 7 days from 5 days after implantation. Values are expressed as the mean ± SD, *p < 0.05 with *t*-test analysis. **B** Images of the tumor weights of sh-RPS27a (*n* = 15) or control (*n* = 16) cell xenografts. *p < 0.05 with *t*-test analysis. **D**, **F**, **H**, **J**, **L**, **N**, **P** IHC analysis of RPS27a, Ki-67, p53, MMP-9, MDM2, E-cadherin, p53, MDM2 and RPL11 in A549 cell implantation, scale bars = 50 μm (magnification, 400×). **E**, **G**, **I**, **K**, **M**, **O**, **Q** Average optical density per area (AOD) (Integral optical density/Area) was performed with digital image analysis for quantification of proteins; *p < 0.05 and ***p < 0.001 with *t*-test analysis (*n =* 4). **R** RPS27a knockdown enhanced the binding of RPL11 and MDM2, thereby leading to p53 activation. The dotted lines represent the weakened interaction, the solid lines represent the enhanced interaction. LUAD, lung adenocarcinoma; NC, negative control; KD, knockdown; IHC, immunohistochemistry

A549 cells with stable knockdown of RPS27a and negative control were injected into the left forelimb muscle in female BALB/c nude mice to explore the effects of RPS27a in cell proliferation and apoptosis; tumor nodules were harvested 47 days after injection (Fig. 8A). Silencing RPS27a inhibited tumor formation (volume and weight) in vivo (Fig. 8B, C). A relatively weak intensity of RPS27a (Fig. 8D, E), Ki-67 (Fig. 8F, G) and MMP-9 (Fig. 8H, I) staining was observed with RPS27a knockdown of xenograft tumor tissue, and a strong intensity of E-cadherin (Fig. 8J, K), p53 (Fig. 8L, M), MDM2 (Fig. 8N, O) and RPL11 (Fig. 8P, Q) was observed with RPS27a knockdown of xenograft tumor tissue. These results indicated that p53 increases apoptosis by ablating RPS27a and inhibits A549 xenograft formation in nude mice.

## Discussion

RPS27a, an ribosomal protein constituting the 40S small subunit of the ribosome, plays an important role in ribosome biogenesis [45]. RPS27a is overexpressed in chronic myeloid leukemia; colon, renal, breast cancers and LUAD [46]. We found that ablation of RPS27a expression induced cell cycle arrest and apoptosis of A549 cells, knockdown of RPS27a increased the expression of RPL11 and promoted the binding of RPL11 to MDM2, thus leading to p53 activation. Therefore, RPS27a is a key factor in maintaining normal levels of p53 through the RPL11–MDM2–p53 pathway in LUAD. In addition, it is crucial in negatively regulating apoptosis in LUAD.

p53 is critical for regulating cell apoptosis and proliferation [47, 48]. The activation of p53 is strictly regulated by its target gene product, the E3 ubiquitin ligase MDM2, thus forming an MDM2-p53 feedback loop [12, 14]. Previous studies have shown that RPs regulate p53 activation by inhibiting MDM2 activity, thereby affecting cell cycle progression and apoptosis [14]. This process is involved in regulating MDM2 binding by RPs, thereby indirectly affecting the negative feedback loop of MDM2-p53 [15]. Overexpressed RPs, such as RPS7 [18], RPL23 [19] and RPL26 [22], have similar functions to RPL11 and RPL5; they bind the central acid domain of MDM2 and subsequently inhibit MDM2-mediated p53 ubiquitination and degradation, thus leading to p53 stabilization. In fact, some weakly expressed RPs, such as RPL22 [22], RPL4 [25] and RPS14 [49], also activate p53 through a process involving the participation of RPL11 and RPL5. The RPL5/RPL11-MDM2-p53 ternary complex is the classical model of RPs and p53 binding, and RPL11 and RPL5 act as nucleolar stress effectors and sensors [50]. RPL5 and RPL11 can bind MDM2 alone or can interact with 5S rRNA, forming the 5S ribonucleoprotein complex (5S RNP), which binds MDM2 and stabilizes p53 [51]. Thus, RPL5 and RPL11 are positive regulators of p53 and act as tumor suppressors [52]. Our previous study showed that endogenous RPL27a and RPL5 interact. Moreover, we found that knockdown of RPL27a increases the interaction of RPL5 and MDM2, and consequently regulates p53 activation in GC-1 cells [53]; thus, RPs may interact with RPL5 and RPL11, thereby regulating p53. Therefore, the potential interaction of RPs with RPL11 and RPL5 in p53 activation cannot be ignored, and RPS27a knockdown may have the aforementioned roles.

RPS27a is overexpressed in renal, breast and colon carcinomas [54, 55], and its gene expression has been found to be markedly elevated in an oncomouse model of hepatocellular carcinoma [56]. It also has an essential role in the activation of cellular checkpoints via p53 [57]. The cell cycle arrest and apoptosis caused by RPS27a knockdown were found to be RPL11 and p53 dependent. Co-transfection experiments demonstrated that the knockdown of p53 eliminated the inhibition of cell viability, cell cycle arrest and apoptosis caused by the decreased expression of RPS27a in A549 cells, thus indicating that these effects were p53 dependent. In addition, the activation of p53 after knockdown of RPS27a was RPL11 dependent, because RPL11 knockdown eliminated the activation of p53 caused by the knockdown of RPS27a and attenuated the increased cell cycle arrest and apoptosis, and inhibition of cell viability induced by RPS27a knockdown.

This study indicated that the knockdown of RPS27a did not disrupt nucleoli in A549 cells, in agreement with the finding that knockdown of RPS6 has no effect on the integrity of the nucleolus [24]. However, knockdown of RPS27a impaired 40S ribosome biogenesis, because the polysome profile results showed that the peak of 40S decreased, and the 80S:40S ratio increased, after RPS27a-siRNA treatment. The RPL11 mRNA levels were not significantly changed, but the RPL11 protein levels were greater in RPS27a-siRNA-treated cells than NC cells, in agreement with the previous finding that knockdown of RPS6 increases RPL11 protein levels, but not mRNA levels [24].

Several studies have demonstrated that inhibition of 40S ribosome biogenesis leads increases ribosome free RPL11, which binds and inhibits MDM2 [24]. Knockdown of RPS6 increased both RPL11, p53 and MDM2 levels, and elevated RPL11 strongly inhibited ubiquitination of both p53 and MDM2, and also inhibited MDM2-mediated p53 ubiquitination [20]. The increased RPL11 protein levels might arise from the disruption of small subunits of ribosomes, thus causing most of the RPL11 transcripts to be recruited to actively translating polysomes and consequently enhancing translation of RPL11 through a 5′-TOP-mediated translation mechanism [24]. Thus, knockdown of RPS27a disrupts the 40S ribosome biogenesis that enhances the RPL11 expression and interaction between RPL11 and MDM2. Therefore, although nucleolar disruption is not a prerequisite for p53 activation after inhibition of 40S ribosome biogenesis induced by RPS27a knockdown, increased RPL11 protein levels are responsible for p53 activation, because the p53 activation after knockdown of RPS27a was RPL11 dependent.

We constructed the protein structures of RPS27a and RPL11 with homology modeling methods, and the protein–protein docking revealed that RPS27a and RPL11 form a stable composite structure. Then, experiments with GST and overexpression plasmids of RPS27a further confirmed that RPS27a binds RPL11, and overexpression of RPS27a enhances this interaction. Subsequently, knockdown of RPS27a weakened the binding of RPS27a and RPL11, but enhanced the binding of RPL11 and MDM2, thereby inhibiting the ubiquitination and degradation of p53 by MDM2.

Previous studies have shown the roles of RPS7 [40], RPS14 [19] and RPS25 [29] in stress-induced p53 activation, and indicated that these RPs are essential for the regulation of p53 in response to ribosomal stress. However, the results of this study are consistent with the effects of RPS26 knockdown on p53 stabilization under stress, but contrast with findings showing that RPS26 regulates p53 transcriptional activity in response to DNA damage [20]. A low dose of ActD destroyed the nucleoli and induced ribosomal stress, thus resulting in the release of nucleolar resident proteins such as RPL5 and RPL11 to the nucleoplasm, where they play a role in p53 activation [58]. Overexpression of these proteins inhibited MDM2-mediated p53 degradation, but ablation of these proteins attenuated the p53 response to low dose ActD [29]. Previous studies have demonstrated that knockdown of RPL11 attenuates the effects of low dose ActD-induced p53 stabilization [59]. In this study, under ActD treatment, knockdown of RPS27a had minimal effects on p53 protein levels and stability, and RPS27a was not found to participate in DNA damage–induced p53 stabilization, these findings may be associated with an increase in RPL11 induced by RPS27a knockdown. The effect of RPS27a knockdown on p53 activation under stress might be strengthened by the increase in RPL11 induced by RPS27a knockdown, because the interaction of MDM2 and RPL11 was enhanced, thereby inhibiting MDM2 E3 ubiquitin ligase activity, stabilizing p53, activating p53 transcriptional activity and inhibiting the cellular functions of A549 cells. The reason for the discrepancy regarding RPS27a and RPS26 in the activation of the p53’transcription response to DNA damage requires further investigation in future studies.

Several RPs, including RPS7 [12], RPS14 [19], RPS26 [20], and RPS25 [29] and RPS2 [52], have been demonstrated to be substrates of MDM2, thus indicating mutual regulation between RPs and MDM2. Similar to other RPs, RPS27a has been demonstrated to be a physiological substrate of MDM2 [60]. RPS27a also binds MDM2, thus inhibiting MDM2 E3 ubiquitin ligase activity and leading to p53 stabilization [60]. The present study focused on the role of RPS27a knockdown in p53 activation through enhancing the binding of RPL11 and MDM2, thereby inhibiting MDM2 E3 ubiquitin ligase activity and leading to p53 stabilization. Therefore, our findings, provide new insights indicating that, beyond the RPs–MDM2–p53 pathway, RPs interact with RPL11, thereby regulating p53. Thus, the RP-RPL5−/RPL11-mediated p53 surveillance system plays an important regulatory role in the progression of cancer.

## Conclusions

In summary, this study is novel in demonstrating that RPS27a binds RPL11 and regulates p53 activation (Fig. 8R). Knockdown of RPS27a induced p53-dependent cell cycle arrest, apoptosis and inhibition of cell viability in A549 cells, in a manner dependent on RPL11. RPS27a directly bound RPL11, and RPS27a knockdown enhanced the binding of RPL11 and MDM2, thereby inhibiting MDM2-mediated p53 ubiquitination and degradation. RPS27a serves as an important regulator of p53 activation by enhancing the interaction of RPL11 and MDM2. Therefore, RPS27a might be a potential target in the treatment of LUAD.

## Supplementary Information

**Additional file 1: Figure S1.** The efficiency of knockdown of RPS27a, RPL11 and p53 by three different siRNAs.

**Additional file 2: Figure S2.** Flow cytometry analysis revealed that the knockdown of RPS27a accelerated G1/S cell cycle progression.

**Additional file 3: Figure S3.** Flow cytometry analysis revealed that the knockdown of RPS27a promoted cell apoptosis.

**Additional file 4: Figure S4.** The knockdown of p53 eliminated RPS27a knockdown-induced apoptosis.

**Additional file 5: Figure S5.** The knockdown of p53 eliminated RPS27a knockdown-accelerated G1/S cell cycle progression.

**Additional file 6: Figure S6.** The knockdown of RPL11 eliminated RPS27a knockdown-induced apoptosis.

**Additional file 7: Figure S7.** The knockdown of RPL11 eliminated RPS27a knockdown-induced accelerated G1/S cell cycle progression.

**Additional file 8: Figure S8.** The A549 cells with stable knockdown of RPS27a were observed under a fluorescence microscope.

**Additional file 9: Figure S9.** The expression of RPS27a in the A549 cells with stable knockdown of the RPS27a.

**Additional file 10: Supplementary file 1.** Plasmid information for RPL11 and RPS27a.

**Additional file 11: Supplementary file 2.** Information on IC_50_ of Dox-treated A549 cells.

**Additional file 12: Supplementary file 3.** Sequences of three different siRNAs for RPL11, RPS27a and p53.

**Additional file 13: Supplementary file 4.** Vector information for RPS27a.

**Additional file 14: Supplementary file 5.** Information on identified proteins.

**Additional file 15: Supplementary file 6.** The correlation of relative expression of RPS27a and apoptotic ratio in A549 cells after CIR.

## Data Availability

The datasets used and/or analyzed during the current study are available from the corresponding author on reasonable request.

## Abbreviations

DAPI: 4', 6-diamidino-2-phenylindole
RPs: Ribosomal proteins
PBS: Phosphate buffered saline
PVDF: Polyvinylidene difluoride
PCR: Polymerase chain reaction
