# Supplementary material for: Loss of RPS27a expression regulates the cell cycle, apoptosis, and proliferation via the RPL11-MDM2-p53 pathway in lung adenocarcinoma cells
Source: J Exp Clin Cancer Res. 2022 Jan 24;41:33. doi: 10.1186/s13046-021-02230-z (PMC8785590; doi:10.1186/s13046-021-02230-z)
Supplement: Supplementary file 1 — Additional file 1: Figure S1. The efficiency of knockdown of RPS27a, RPL11 and p53 by three different siRNAs. [file 13046_2021_2230_MOESM1_ESM.doc]

**
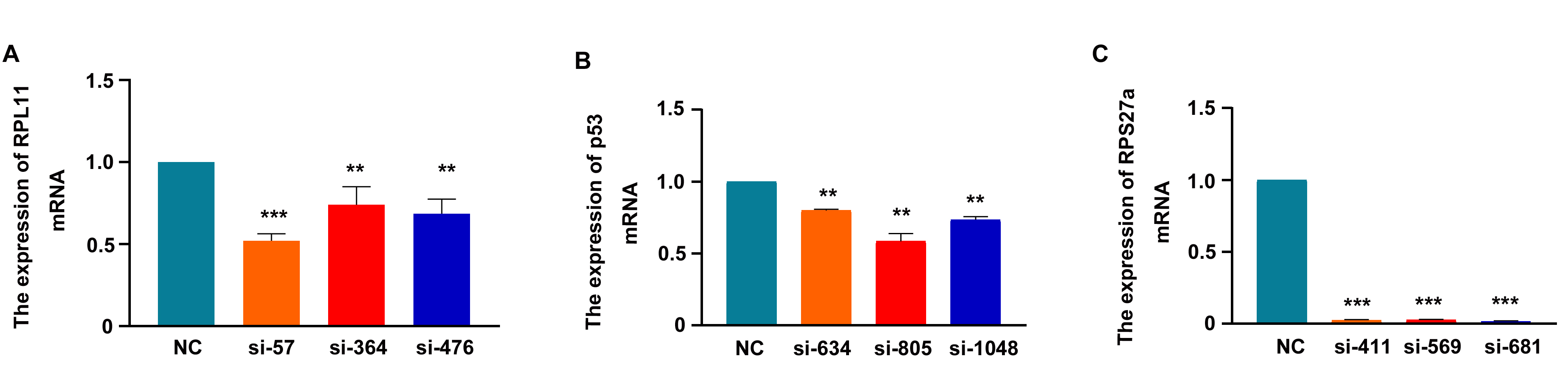
**

**Figure S1.** The efficiency of knockdown of RPS27a, RPL11 and p53 in A549 cells by three different siRNAs using real-time PCR. (A) The expression of RPL11 mRNA. (B) The expression of p53 mRNA. (C) The expression of RPS27a mRNA. Statistical analysis was performed using one-way analysis of variance (ANOVA) with Turkey and compared with NC (***P* < 0.05; ***P* < 0.01). NC, negative control. The number of each gene represents the each siRNA sequence.
