# Supplementary material for: Loss of RPS27a expression regulates the cell cycle, apoptosis, and proliferation via the RPL11-MDM2-p53 pathway in lung adenocarcinoma cells
Source: J Exp Clin Cancer Res. 2022 Jan 24;41:33. doi: 10.1186/s13046-021-02230-z (PMC8785590; doi:10.1186/s13046-021-02230-z)
Supplement: Supplementary file 2 — Additional file 2: Figure S2. Flow cytometry analysis revealed that the knockdown of RPS27a accelerated G1/S cell cycle progression. [file 13046_2021_2230_MOESM2_ESM.doc]

**
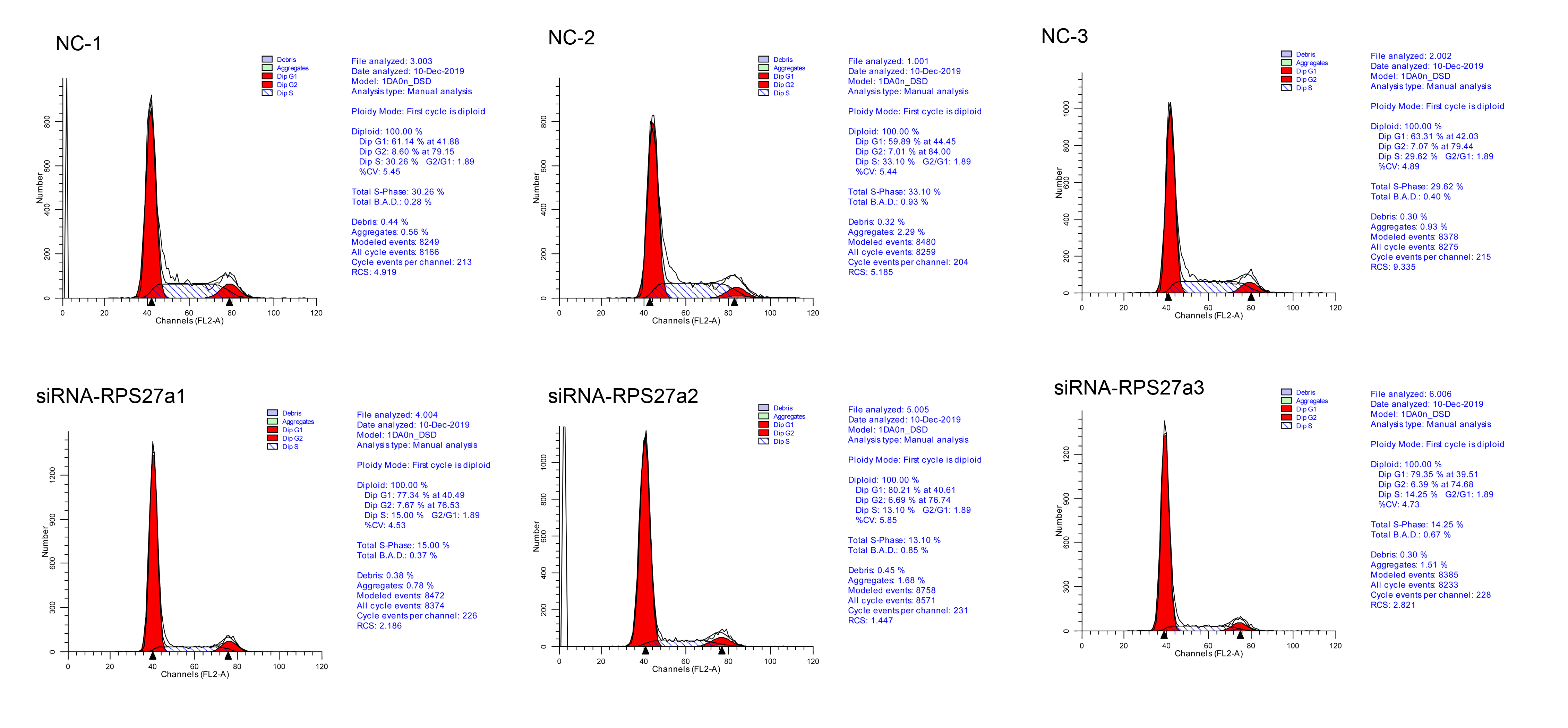
**

**Figure S2.** Flow cytometry analysis revealed that the knockdown of RPS27a increased G1-phase arrest in A549 cells.
