# Supplementary material for: Loss of RPS27a expression regulates the cell cycle, apoptosis, and proliferation via the RPL11-MDM2-p53 pathway in lung adenocarcinoma cells
Source: J Exp Clin Cancer Res. 2022 Jan 24;41:33. doi: 10.1186/s13046-021-02230-z (PMC8785590; doi:10.1186/s13046-021-02230-z)
Supplement: Supplementary file 3 — Additional file 3: Figure S3. Flow cytometry analysis revealed that the knockdown of RPS27a promoted cell apoptosis. [file 13046_2021_2230_MOESM3_ESM.doc]

**
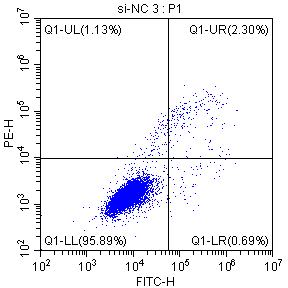

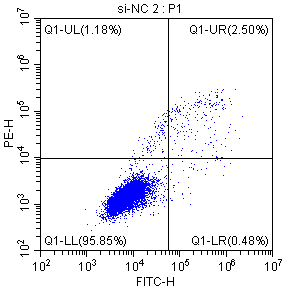

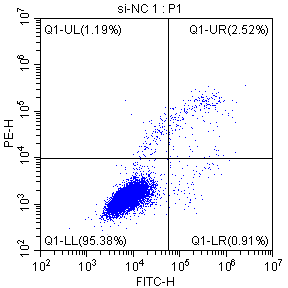
**

**
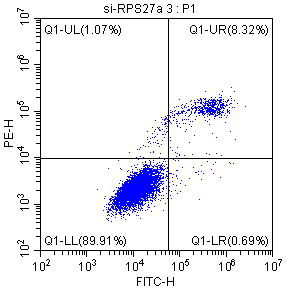

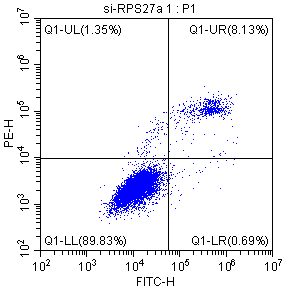
**

**
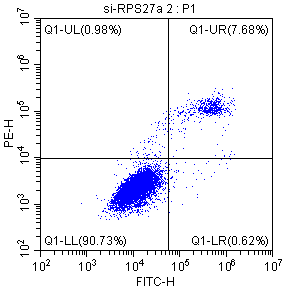
**

**Figure S3.** Flow cytometry analysis revealed that the knockdown of RPS27a promoted apoptosis in A549 cells.
