# Supplementary material for: Loss of RPS27a expression regulates the cell cycle, apoptosis, and proliferation via the RPL11-MDM2-p53 pathway in lung adenocarcinoma cells
Source: J Exp Clin Cancer Res. 2022 Jan 24;41:33. doi: 10.1186/s13046-021-02230-z (PMC8785590; doi:10.1186/s13046-021-02230-z)
Supplement: Supplementary file 4 — Additional file 4: Figure S4. The knockdown of p53 eliminated RPS27a knockdown-induced apoptosis. [file 13046_2021_2230_MOESM4_ESM.doc]

| 1. A549 si-NC | | |
| --- | --- | --- |
| 01 | 02 | 03 |
| 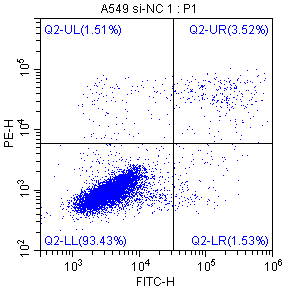 | 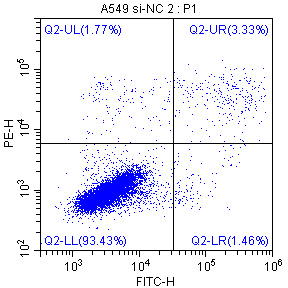 | 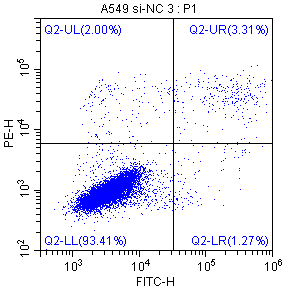 |
| 2.A549 si-p53 | | |
| 01 | 02 | 03 |
| 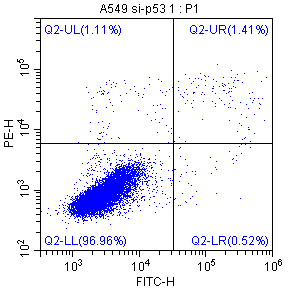 | 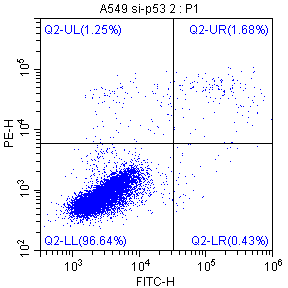 | 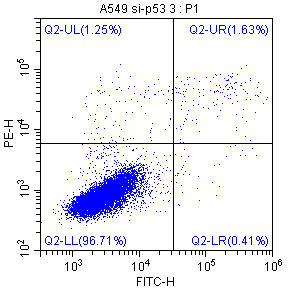 |
| 3.A549 si-RPS27a | | |
| 01 | 02 | 03 |
| 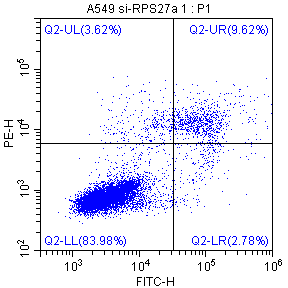 | 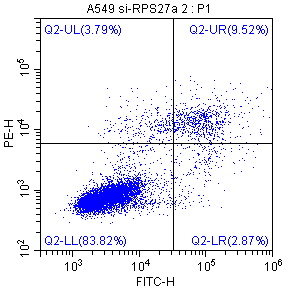 | 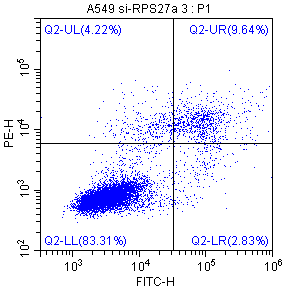 |
| 4.A549 si-p53+si-RPS27a | | |
| 01 | 02 | 03 |
| 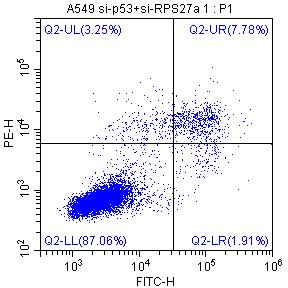 | 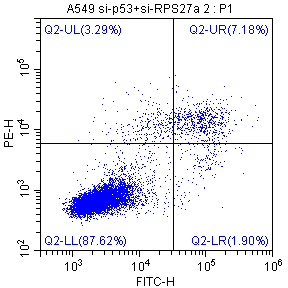 | 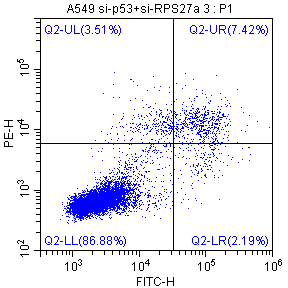 |
| Blank | FITC | PI |
| 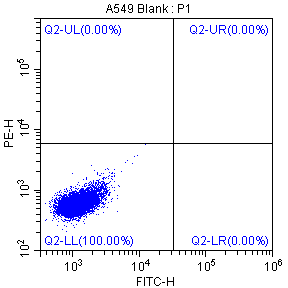 | 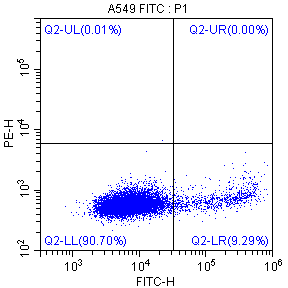 | 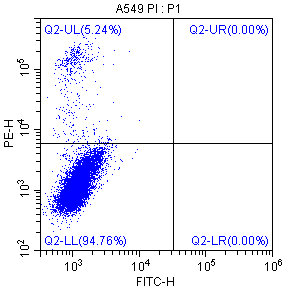 |
| 5. H1299 si-NC | | |
| 01 | 02 | 03 |
| 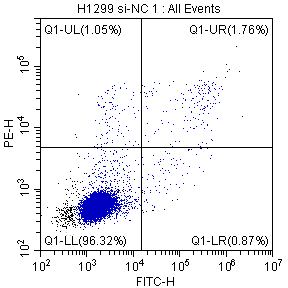 | 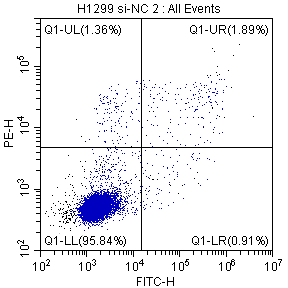 | 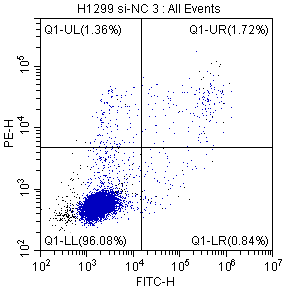 |
| 6.H1299 si-RPS27a | | |
| 01 | 02 | 03 |
| 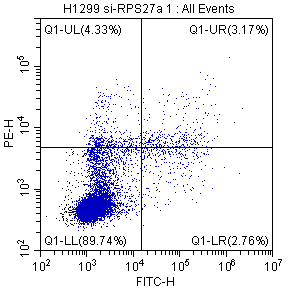 | 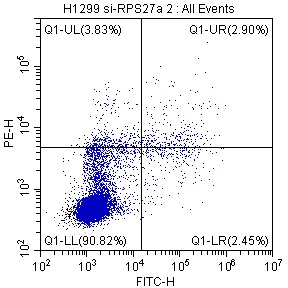 | 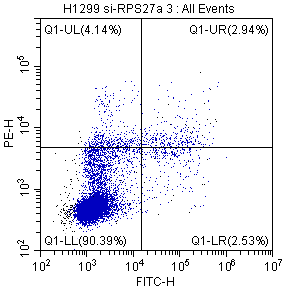 |
| Blank | FITC | PI |
| 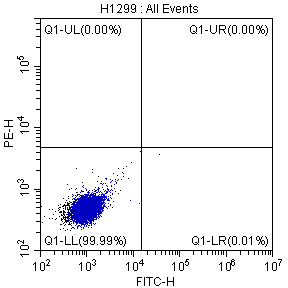 | 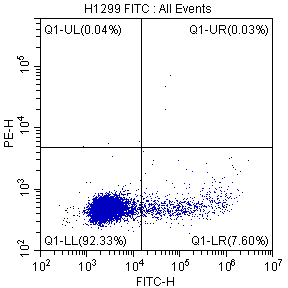 | 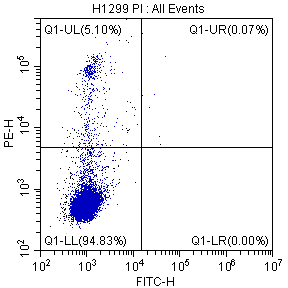 |

**Figure S4.** The knockdown of p53 eliminated the knockdown of RPS27a induced apoptosis.
