# Supplementary material for: Loss of RPS27a expression regulates the cell cycle, apoptosis, and proliferation via the RPL11-MDM2-p53 pathway in lung adenocarcinoma cells
Source: J Exp Clin Cancer Res. 2022 Jan 24;41:33. doi: 10.1186/s13046-021-02230-z (PMC8785590; doi:10.1186/s13046-021-02230-z)
Supplement: Supplementary file 5 — Additional file 5: Figure S5. The knockdown of p53 eliminated RPS27a knockdown-accelerated G1/S cell cycle progression. [file 13046_2021_2230_MOESM5_ESM.doc]

| 1. A549 si-NC | | |
| --- | --- | --- |
| 01 | 02 | 03 |
| 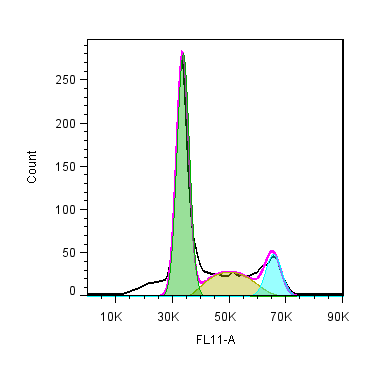 | 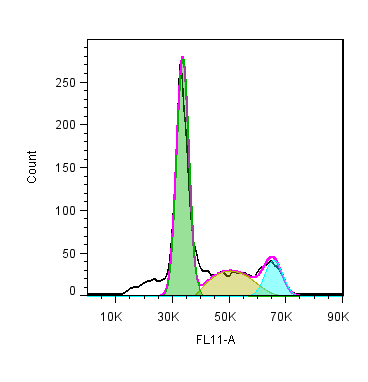 | 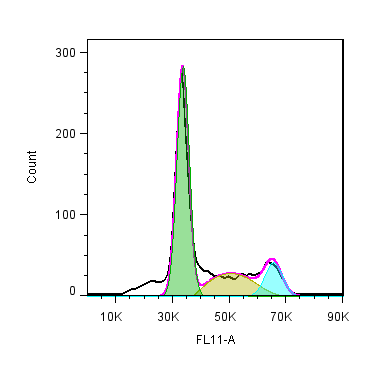 |
| Freq. G1 = 59.42  Freq. S = 20.17  Freq. G2 = 13.24 | Freq. G1 = 58.72  Freq. S = 19.14  Freq. G2 = 12.92 | Freq. G1 = 59.4  Freq. S = 19.22  Freq. G2 = 12.5 |
| 1. A549 si-p53 | | |
| 01 | 02 | 03 |
| 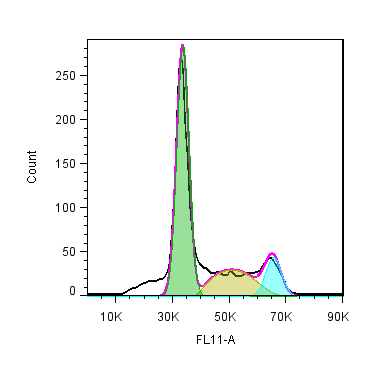 | 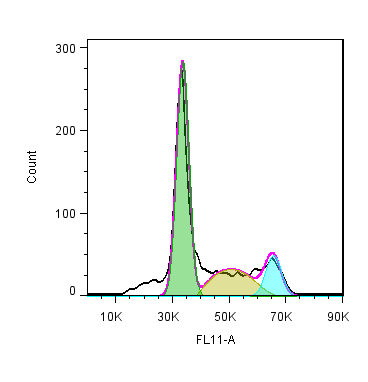 | 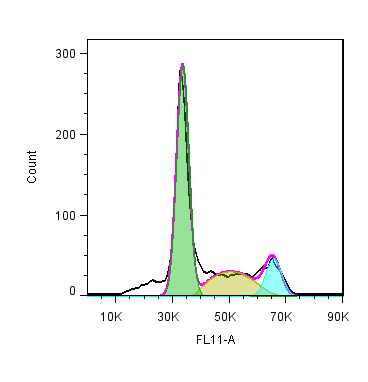 |
| Freq. G1 = 59.58  Freq. S = 19.05  Freq. G2 = 11.87 | Freq. G1 = 57.96  Freq. S = 19.05  Freq. G2 = 12.95 | Freq. G1 = 58.69  Freq. S = 20.28  Freq. G2 = 12.2 |
| 3. A549 si-RPS27a | | |
| 01 | 02 | *03* |
| 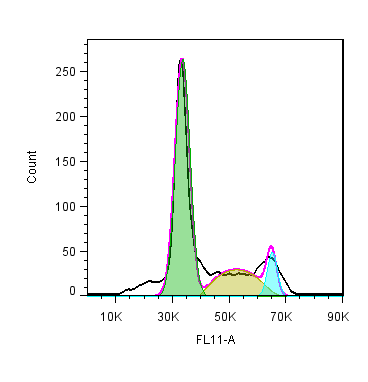 | 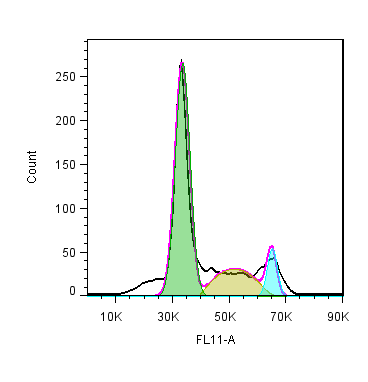 | 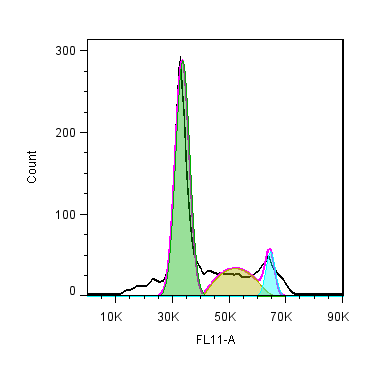 |
| Freq. G1 = 65.69  Freq. S = 17.89  Freq. G2 = 8.33 | Freq. G1 = 65.39  Freq. S = 17.09  Freq. G2 = 9.12 | Freq. G1 = 66.88  Freq. S = 14.45  Freq. G2 = 8.4 |
| 4. A549 si-p53+si-RPS27a | | |
| 01 | 02 | *03* |
| 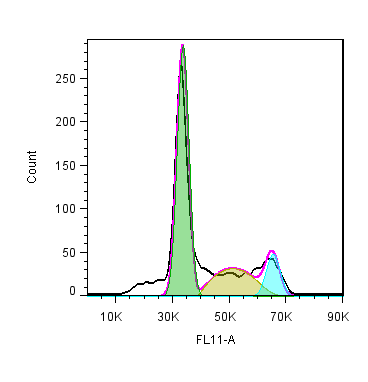 | 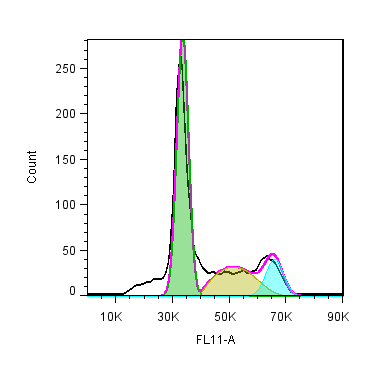 | 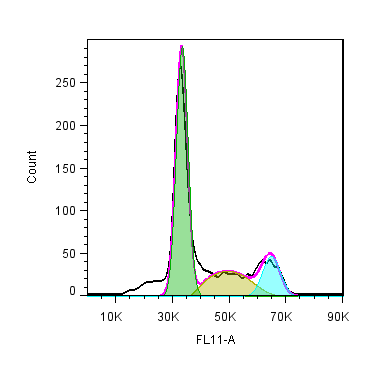 |
| Freq. G1 = 57.69  Freq. S = 19.11  Freq. G2 = 11.52 | Freq. G1 = 57.2  Freq. S = 18.51  Freq. G2 = 12.34 | Freq. G1 = 57.28  Freq. S = 19.58  Freq. G2 = 13.89 |
| 5.H1299 si-NC | | |
| 01 | 02 | *03* |
| 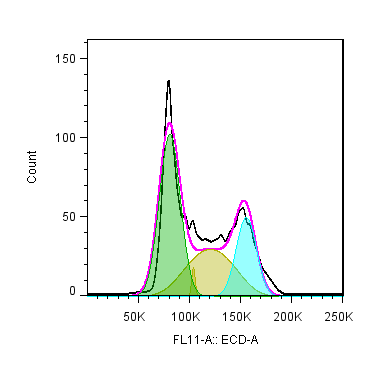 | 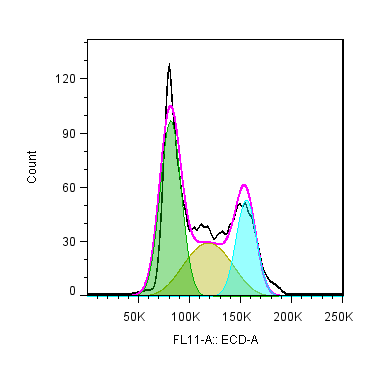 | 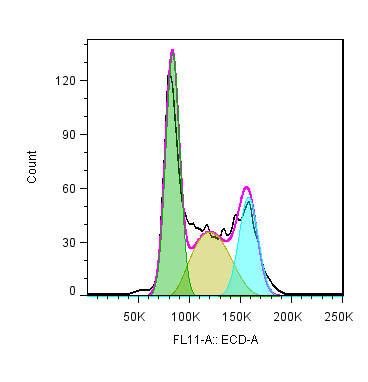 |
| Freq. G1 = 47.1  Freq. S = 32.48  Freq. G2 = 22.21 | Freq. G1 = 46.23  Freq. S = 31.02  Freq. G2 = 24.52 | Freq. G1 = 43.11  Freq. S = 30.02  Freq. G2 = 23.92 |
| 6. H1299 si-RPS27a | | |
| 01 | 02 | *03* |
| 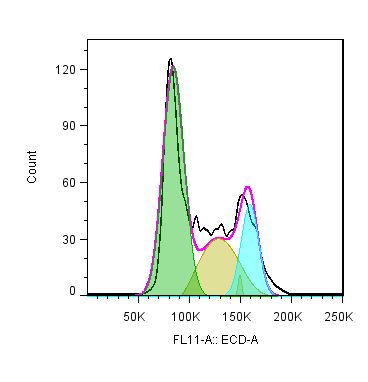 | 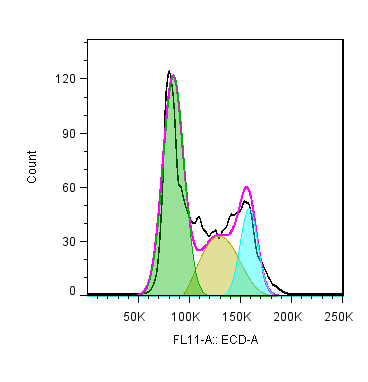 | 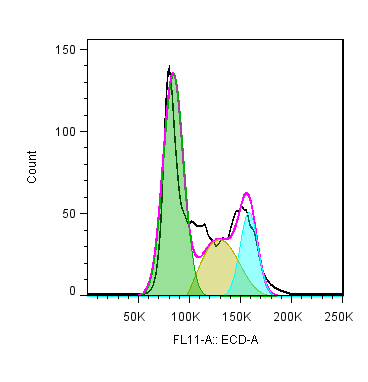 |
| Freq. G1 = 57.24  Freq. S = 24.85  Freq. G2 = 19.22 | Freq. G1 = 56.93  Freq. S = 25.1  Freq. G2 = 19.09 | Freq. G1 = 59.09  Freq. S = 22.5  Freq. G2 = 19.48 |

**Figure S5.** The knockdown of p53 eliminated the knockdown of RPS27a increased G1-phase arrest
