# Supplementary material for: Loss of RPS27a expression regulates the cell cycle, apoptosis, and proliferation via the RPL11-MDM2-p53 pathway in lung adenocarcinoma cells
Source: J Exp Clin Cancer Res. 2022 Jan 24;41:33. doi: 10.1186/s13046-021-02230-z (PMC8785590; doi:10.1186/s13046-021-02230-z)
Supplement: Supplementary file 6 — Additional file 6: Figure S6. The knockdown of RPL11 eliminated RPS27a knockdown-induced apoptosis. [file 13046_2021_2230_MOESM6_ESM.doc]

| 1. si-NC | | |
| --- | --- | --- |
| 01 | 02 | 03 |
| 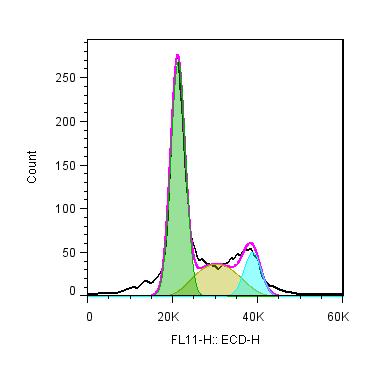 | 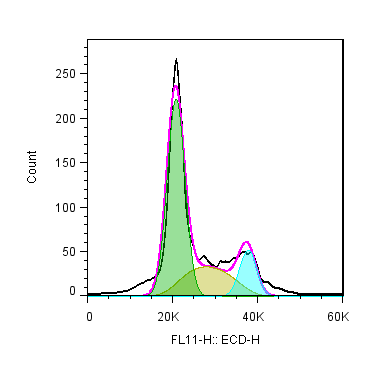 | 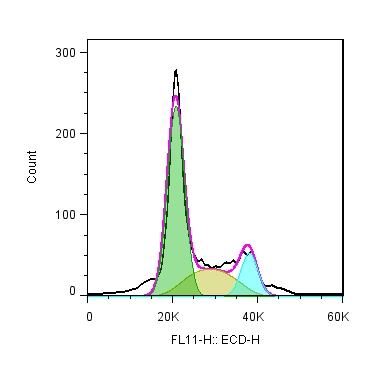 |
| Freq. G1 = 57.41%  Freq. S = 23.31%  Freq. G2 = 12.59% | Freq. G1 = 58.27%  Freq. S = 23.79%  Freq. G2 = 13.14% | Freq. G1 = 58.58%  Freq. S = 23.62%  Freq. G2 = 13.23% |
| 1. si-RPL11 | | |
| 01 | 02 | 03 |
| **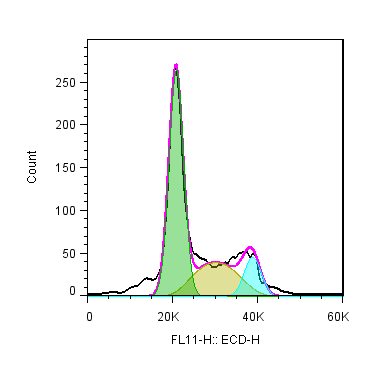** | 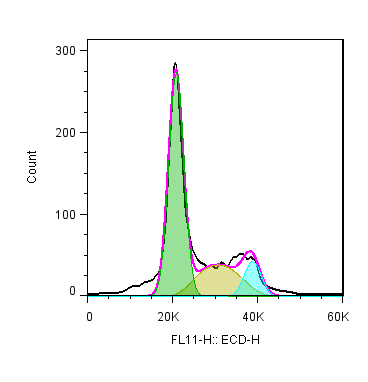 | 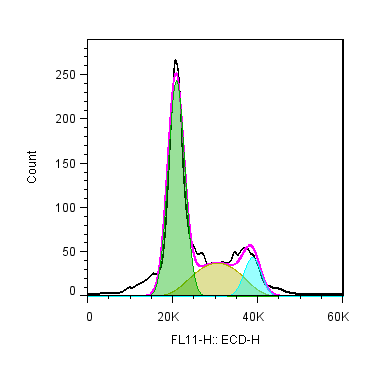 |
| Freq. G1 = 56.56%  Freq. S = 25.26%  Freq. G2 = 11.48% | Freq. G1 = 59.98%  Freq. S = 23.34%  Freq. G2 = 10.46% | Freq. G1 = 58.38%  Freq. S = 25.58%  Freq. G2 = 10.89% |
| 3.si-RPS27a | | |
| 01 | 02 | *03* |
| 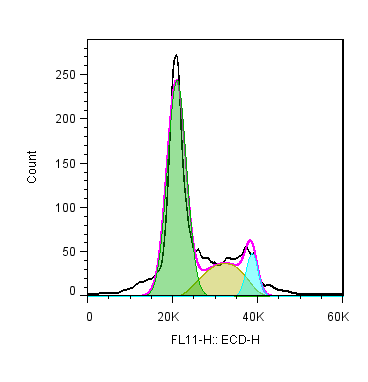 | 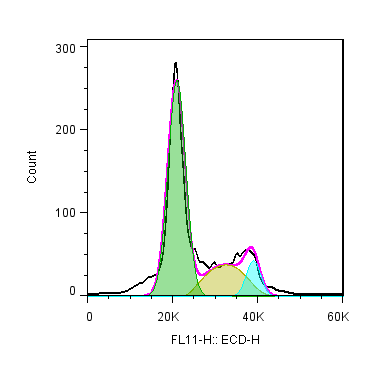 | *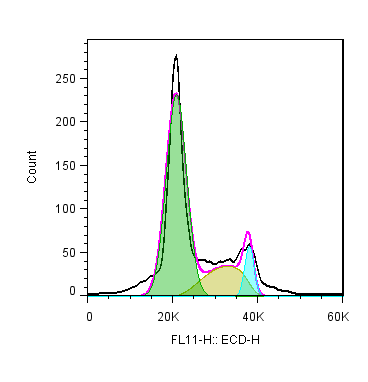* |
| Freq. G1 = 66.41%  Freq. S = 20.73%  Freq. G2 = 8.29% | Freq. G1 = 66.06%  Freq. S = 20.29%  Freq. G2 = 8.89% | Freq. G1 = 67.34%  Freq. S = 19.11%  Freq. G2 = 7.72% |
| 4.si-RPL11+si-RPS27a | | |
| 01 | 02 | *03* |
| 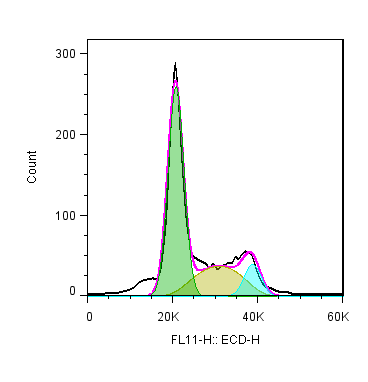 | 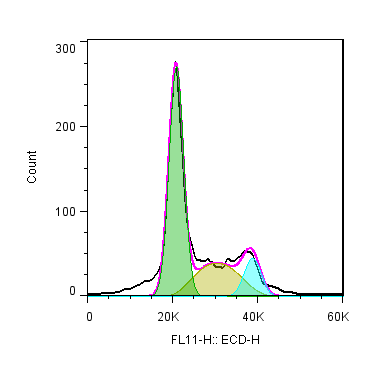 | *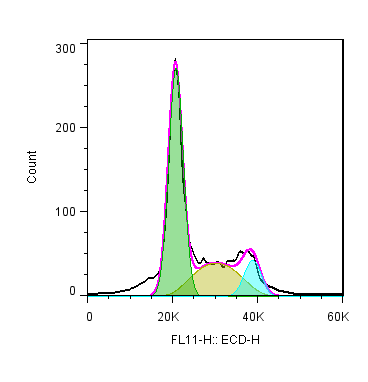* |
| Freq. G1 = 59.84%  Freq. S = 25.47%  Freq. G2 = 9.87% | Freq. G1 = 57.13%  Freq. S = 24.48%  Freq. G2 = 11.08% | Freq. G1 = 57.05%  Freq. S = 25.97%  Freq. G2 = 10.6% |

**Figure S6.** The knockdown of RPL11 eliminated the knockdown of RPS27a increased G1-phase arrest in A549 cells.
