# Supplementary material for: Loss of RPS27a expression regulates the cell cycle, apoptosis, and proliferation via the RPL11-MDM2-p53 pathway in lung adenocarcinoma cells
Source: J Exp Clin Cancer Res. 2022 Jan 24;41:33. doi: 10.1186/s13046-021-02230-z (PMC8785590; doi:10.1186/s13046-021-02230-z)
Supplement: Supplementary file 8 — Additional file 8: Figure S8. The A549 cells with stable knockdown of RPS27a were observed under a fluorescence microscope. [file 13046_2021_2230_MOESM8_ESM.doc]

**
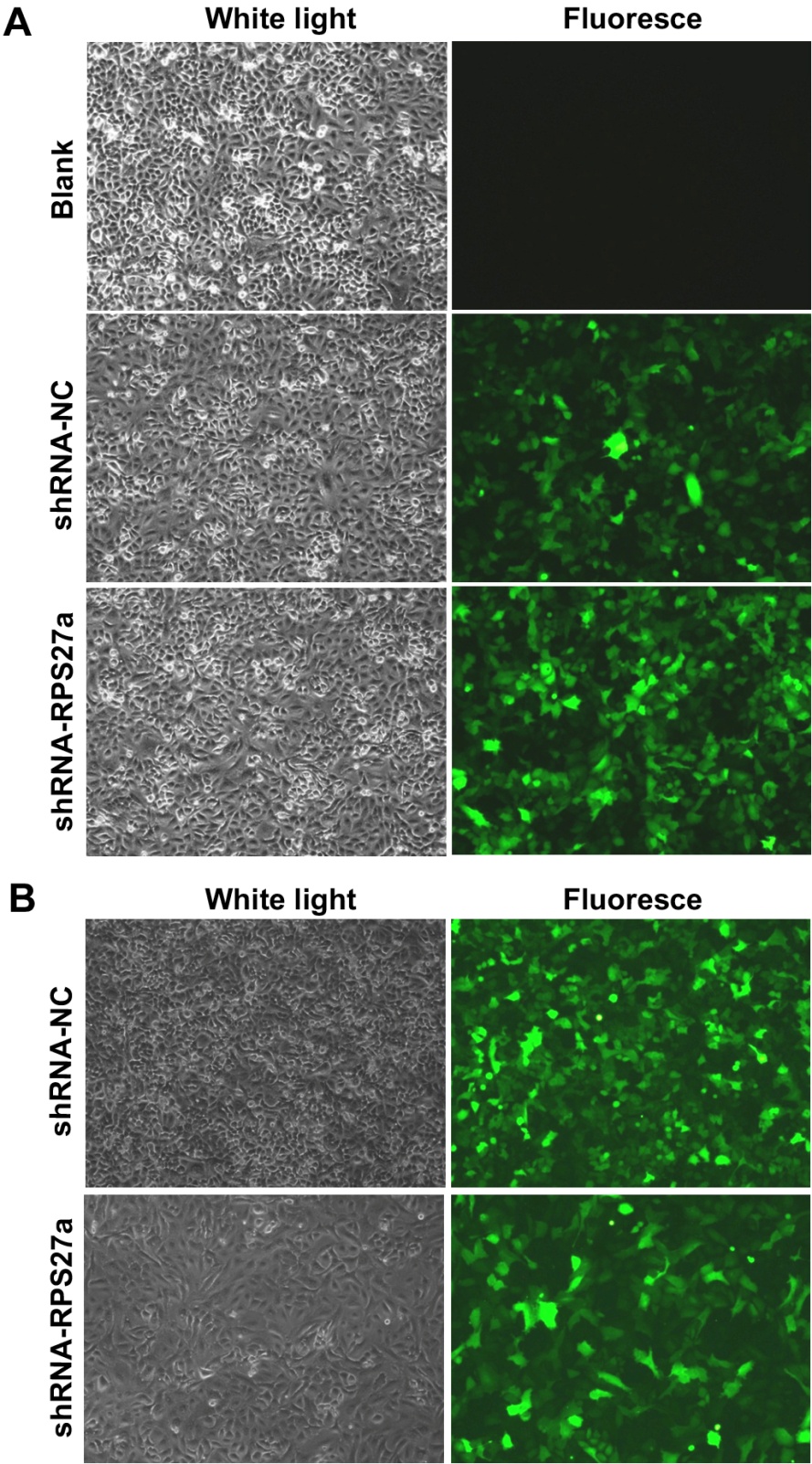
**

**Figure S8.** The A549 cells were observed under a fluorescence microscope and further selected by puromycin with a final concentration of 2 μg/mL 72 h (A) and 14 d (B) after lentiviral infection. Magnification, 1000×; NC, negative control.
