# Supplementary material for: Loss of RPS27a expression regulates the cell cycle, apoptosis, and proliferation via the RPL11-MDM2-p53 pathway in lung adenocarcinoma cells
Source: J Exp Clin Cancer Res. 2022 Jan 24;41:33. doi: 10.1186/s13046-021-02230-z (PMC8785590; doi:10.1186/s13046-021-02230-z)
Supplement: Supplementary file 9 — Additional file 9: Figure S9. The expression of RPS27a in the A549 cells with stable knockdown of the RPS27a. [file 13046_2021_2230_MOESM9_ESM.doc]

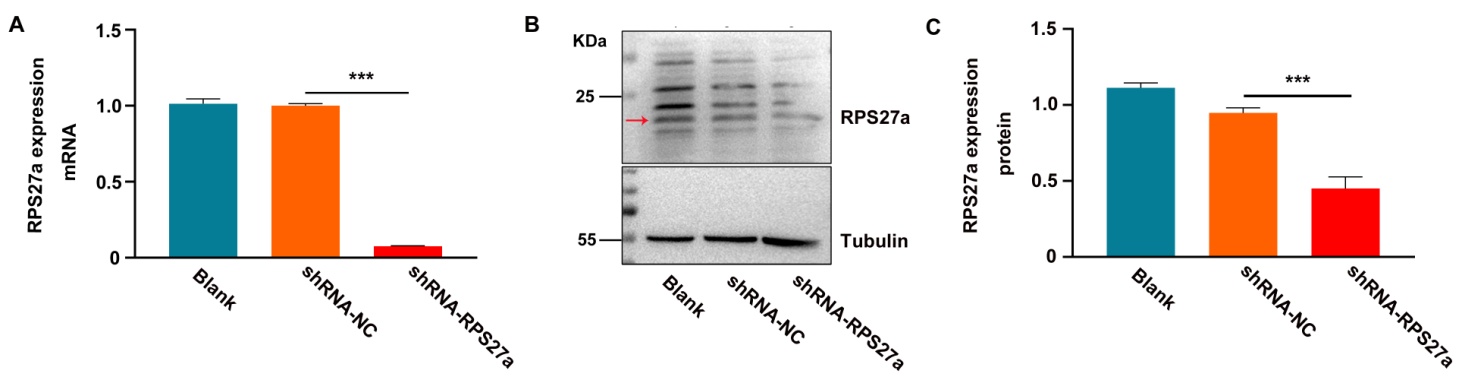
**Figure S9. The expression of RPS27a in the A549 cells with stable knockdown of the RPS27a.** (A) The expression of RPS27a mRNA levels was analyzed by real-time PCR. Error bars represent means ± SD (*n* = 3). Statistical analysis was performed using the Student’s *t*-test (^***^*P* < 0.001). (B) The RPS27a protein levels were detected by immunoblotting. (C) The expression of RPS27a protein levels was analyzed by RPS27a/tubulin. The Error bars represent means ± SD (*n* = 3). Statistical analysis was performed using the Student’s *t*-test (^***^*P* < 0.001).
