# Supplementary figures and images for: Loss of RPS27a expression regulates the cell cycle, apoptosis, and proliferation via the RPL11-MDM2-p53 pathway in lung adenocarcinoma cells
Source: J Exp Clin Cancer Res. 2022 Jan 24;41:33. doi: 10.1186/s13046-021-02230-z (PMC8785590; doi:10.1186/s13046-021-02230-z)

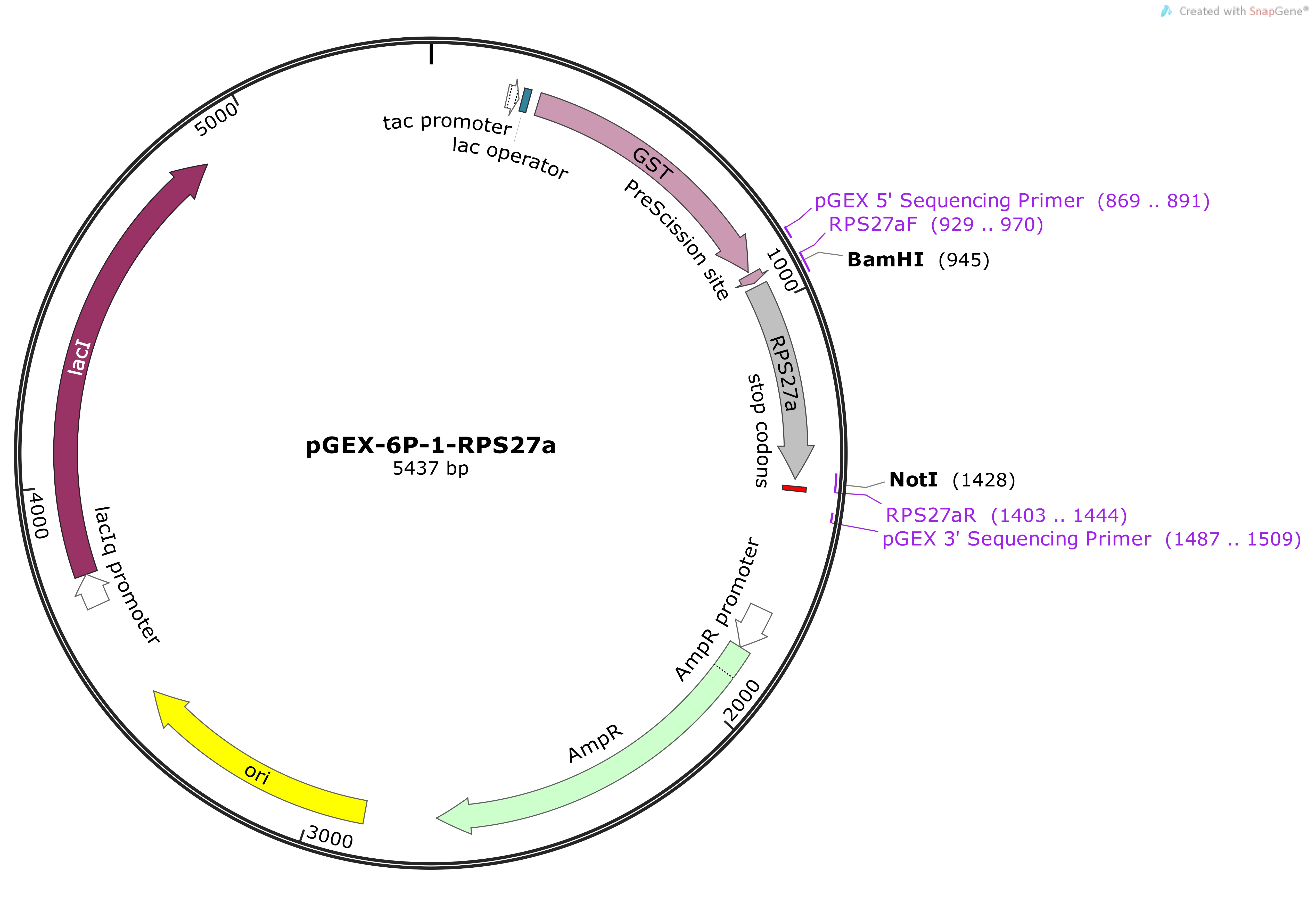


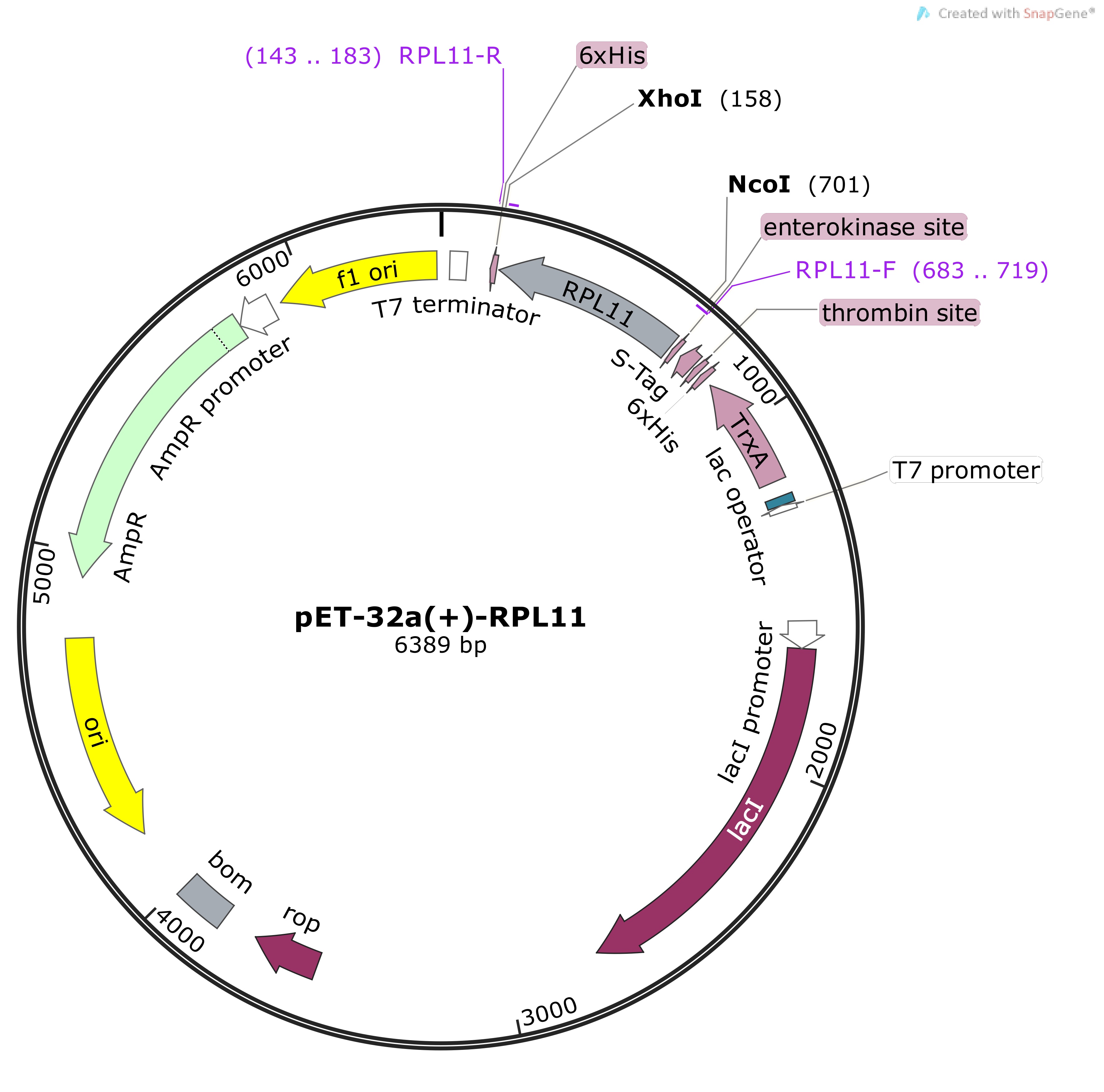


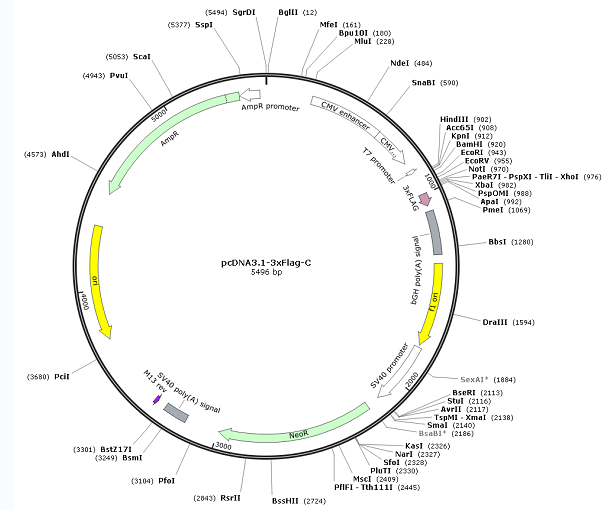

Supplement: Supplementary file 10 — Additional file 10: Supplementary file 1. Plasmid information for RPL11 and RPS27a. [file 13046_2021_2230_MOESM10_ESM.doc]
