# Supplementary material for: Loss of RPS27a expression regulates the cell cycle, apoptosis, and proliferation via the RPL11-MDM2-p53 pathway in lung adenocarcinoma cells
Source: J Exp Clin Cancer Res. 2022 Jan 24;41:33. doi: 10.1186/s13046-021-02230-z (PMC8785590; doi:10.1186/s13046-021-02230-z)
Supplement: Supplementary file 13 — Additional file 13: Supplementary file 4. Vector information for RPS27a. [file 13046_2021_2230_MOESM13_ESM.doc]

| NO. | 5’ | STEM | Loop | STEM | 3’ |
| --- | --- | --- | --- | --- | --- |
| shRPS27a-F | CCGG | GTGCCCTTCTGATGAATGT | TTCAAGAGA | ACATTCATCAGAAGGGCAC | TTTTTTg |
| shRPS27a-R | AATTCAAAAAA | GTGCCCTTCTGATGAATGT | TCTCTTGAA | ACATTCATCAGAAGGGCAC |  |
| shNC-F | CCGG | TTCTCCGAACGTGTCACGT | TTCAAGAGA | ACGTGACACGTTCGGAGAA | TTTTTTG |
| shNC-R | AATTCAAAAAA | TTCTCCGAACGTGTCACGT | TCTCTTGAA | ACGTGACACGTTCGGAGAA |  |

The sequence of RPS27a
