# Supplementary material for: Loss of RPS27a expression regulates the cell cycle, apoptosis, and proliferation via the RPL11-MDM2-p53 pathway in lung adenocarcinoma cells
Source: J Exp Clin Cancer Res. 2022 Jan 24;41:33. doi: 10.1186/s13046-021-02230-z (PMC8785590; doi:10.1186/s13046-021-02230-z)
Supplement: Supplementary file 15 — Additional file 15: Supplementary file 6. The correlation of relative expression of RPS27a and apoptotic ratio in A549 cells after CIR. [file 13046_2021_2230_MOESM15_ESM.doc]

|  | Early apoptotic ratio | Late apoptotic ratio |
| --- | --- | --- |
| Relative expression of RPS27a | 12 h, *r* = 0.500 | 12 h, *r* = -1.000** |
| Relative expression of RPS27a | 24 h, *r* = -0.500 | 24 h, *r* = 1.000** |
| Relative expression of RPS27a | 48 h, *r* = -0.500 | 48 h, *r* = -1.000** |

**Table.1** The correlation of relative expression of RPS27a and apoptotic ratio in A549 cells after CIR were assessed using Spearman,s correlation coefficient. **Differences are indicated at *p* < 0.01.
